# Pathophysiology and genetics of salt-sensitive hypertension

**DOI:** 10.3389/fphys.2022.1001434

**Published:** 2022-09-13

**Authors:** Dina Maaliki, Maha M. Itani, Hana A. Itani

**Affiliations:** ^1^ Department of Pharmacology and Toxicology, Faculty of Medicine, American University of Beirut, Beirut, Lebanon; ^2^ Division of Clinical Pharmacology, Department of Medicine, Vanderbilt University Medical Center, Nashville, TN, United States

**Keywords:** inflammation, hypertension, immunity, salt-sensitive hypertension, kidney injury

## Abstract

Most hypertensive cases are primary and heavily associated with modifiable risk factors like salt intake. Evidence suggests that even small reductions in salt consumption reduce blood pressure in all age groups. In that regard, the ACC/AHA described a distinct set of individuals who exhibit salt-sensitivity, regardless of their hypertensive status. Data has shown that salt-sensitivity is an independent risk factor for cardiovascular events and mortality. However, despite extensive research, the pathogenesis of salt-sensitive hypertension is still unclear and tremendously challenged by its multifactorial etiology, complicated genetic influences, and the unavailability of a diagnostic tool. So far, the important roles of the renin-angiotensin-aldosterone system, sympathetic nervous system, and immune system in the pathogenesis of salt-sensitive hypertension have been studied. In the first part of this review, we focus on how the systems mentioned above are aberrantly regulated in salt-sensitive hypertension. We follow this with an emphasis on genetic variants in those systems that are associated with and/or increase predisposition to salt-sensitivity in humans.

## 1 Introduction

### 1.1 Global burden of CVDs: Contribution of hypertension

The global burden of cardiovascular diseases (CVDs) is a major public health issue, compromising social and economic development worldwide and accounting for 17.9 million deaths annually (World Health Organization, 2021). It is well known that one of the most important risk factors for CVDs is hypertension (HTN) (Lloyd-Jones et al., 2010). HTN, or the silent killer, affects more than 1 billion people worldwide (WHO, 2021). A main manifestation of HTN is end-organ damage, which makes HTN a leading cause of mortality from stroke, heart failure, myocardial infarction, and kidney damage. (Narayan et al., 2010; Casey et al., 2019). In 2017, The American College of Cardiology/American Heart Association (ACC/AHA) set more stringent blood pressure (BP) goals and redefined stage 1 HTN as a sustained BP of 130/80 mm Hg or more (Whelton et al., 2018). The lower cutoffs are supported by large observational studies (Lewington et al., 2002; Rapsomaniki et al., 2014) that demonstrated an important association between BP and CVD risk and randomized controlled trials (RCTs) (Thomopoulos et al., 2014; Wright et al., 2015; Ettehad et al., 2016) that revealed an important benefit of pharmacological treatment in individuals with stage 1 HTN.

### 1.2 Salt-sensitivity definition: Heterogeneity of blood pressure to dietary sodium intake

It is reported that excessive salt intake is responsible for around half of the disease burden ascribed to high BP (World Health Organization, 2012). However, the BP response to salt intake is not uniform among individuals (Elijovich et al., 2016). In this regard, The ACC/AHA identified a category of individuals who demonstrated “salt-sensitivity.” As defined by the ACC/AHA, salt-sensitivity is “a physiological trait present in rodents and other mammals, including humans, in which the BP of some members of the population exhibits changes parallel to changes in salt intake” (Elijovich et al., 2016). In these patients, acute salt loading elicits greater surges in BP, and salt deprivation causes larger drops in BP compared to salt-resistant individuals.

### 1.3 Incidence and predisposing risk factors of salt-sensitive hypertension

Recent findings indicate that reducing salt intake reduces BP and decreases cardiovascular risks (Weinberger et al., 2001; He et al., 2013). Salt-sensitivity affects nearly 50% of the hypertensive and 25% of the normotensive individuals (Weinberger et al., 1986; He et al., 2013; Elijovich et al., 2016), and is an important risk factor for CVD and mortality independently from BP elevation (Weinberger et al., 2001). Because of this, the World Health Organization (WHO) currently recommends that adults reduce sodium intake to less than 5 g of salt/day (2 g of sodium/day) (Marcus et al., 2013; Santos et al., 2021).

Many factors contribute to salt-sensitivity, including genetic background, black race, age, sex, body mass index, and co-morbidities such as HTN, diabetes, kidney disease and metabolic syndrome (Weinberger, 1996; Franco and Oparil, 2006; Elijovich et al., 2016). For example, salt-sensitivity appears to be more common in females and obese individuals (Elijovich et al., 2016).

### 1.4 Salt-sensitivity: Clinical evaluation

Currently, there is no standardized method for diagnosing salt-sensitivity. Failure to accurately identify salt-sensitive individuals greatly impedes advances in determining demographics, clinical relevance, and therapeutic strategies in salt-sensitivity research. In this section, we will discuss the principal experimental approaches that are currently in use to classify salt-sensitive individuals.

According to the ACC/AHA, the methods to diagnose salt-sensitivity are divided into “inpatient-” and “outpatient-” acute protocols. The “outpatient dietary protocol” requires approximately 2 weeks to conduct. It includes a strict low salt diet for 1 week followed by a high salt diet for the second week (Elijovich et al., 2016). BP changes are then mapped to variations in dietary sodium intake. On the other hand, the “inpatient dietary protocol” requires 3 days and consists of rapid extracellular volume (ECV) expansion on the first day with intravenous normal saline loading, which is followed by sodium and volume depletion induced with a low sodium diet in combination with furosemide (Sharma et al., 1994; Galletti et al., 1997; de la Sierra et al., 2002; Gu et al., 2013; Elijovich et al., 2016; Galletti and Strazzullo, 2016; Kurtz et al., 2017a). Currently, the ACC/AHA doesn’t recommend one method over the other, stating that evidence for the superiority of one protocol is inconclusive. However, the outpatient method seems to be preferred among investigators primarily because of its higher reproducibility and its stronger ability to predict cardiovascular risk (Galletti et al., 1997; Morimoto et al., 1997; Gu et al., 2013; Kurtz et al., 2017a; Kurtz et al., 2017b).

**FIGURE 1:**
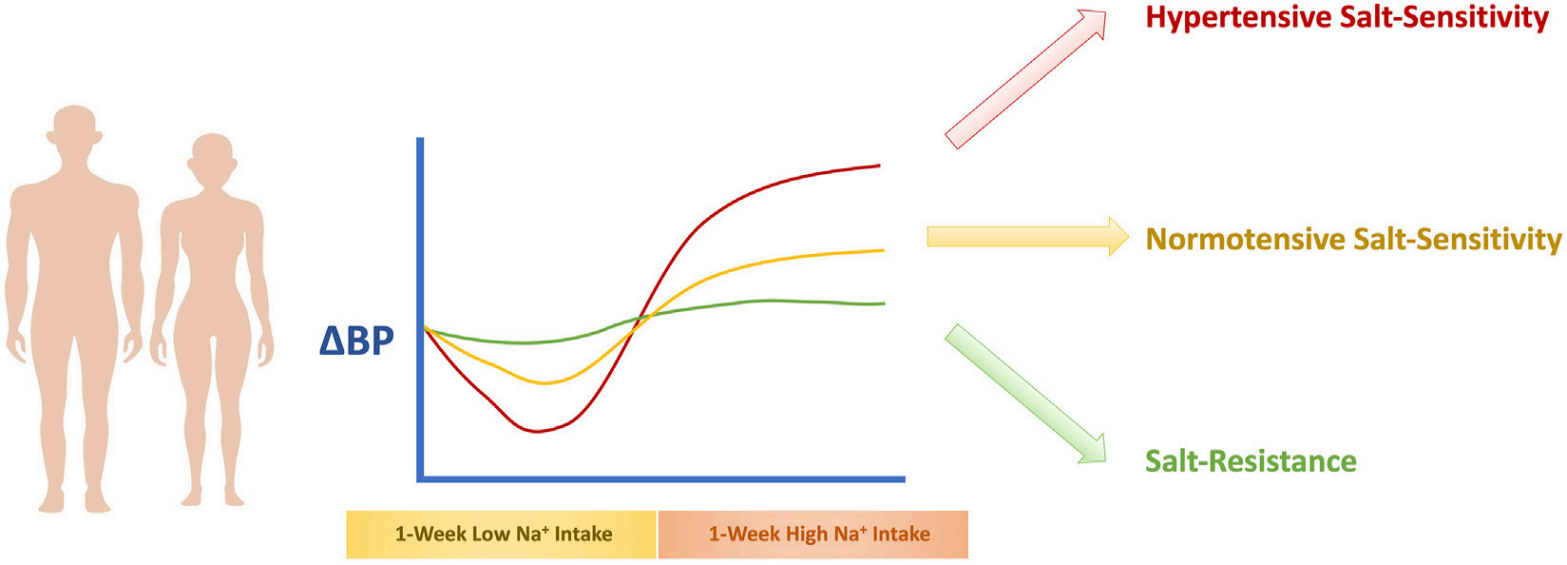
Determination of Salt-Sensitivity of BP. The recommended method to diagnose salt-sensitivity involves exposure to a 1-week high salt diet phase followed by a 1-week low salt diet phase, that is accompanied with BP measurements. If the BP increase is equal to or exceeds 3–5 mmHg, the individual is considered a normotensive salt-sensitive individual. If the BP change is below 3–5 mmHg, the individual is considered salt-resistant. If the BP change is equal to or exceeds 8–10 mm Hg, the individual is classified as a hypertensive salt-sensitive individual.

An important challenge with the diagnosis of salt-sensitivity is the determination of precise cutoff points for the classification of individuals. Currently, the most agreed upon cutoff points are a change in sphygmomanometric readings of mean arterial pressure (MAP) of at least 3–5 mm Hg in response to the change in salt intake for the normotensive salt-sensitive individual (Sharma et al., 1989; Overlack et al., 1993; Schmidlin et al., 2011), and a change in MAP of at least 8–10 mm Hg (Kawasaki et al., 1978; Bigazzi et al., 1994; Draaijer et al., 1995) for the hypertensive salt-sensitive individual. An indeterminate category of a change in MAP between 5–to 9 mmHg is used to allow for greater distinguishability (Grim et al., 1977). In the case of ambulatory BP measurement, the preferred cutoff is at least a 5% change in MAP over 24 h (de la Sierra et al., 2002). Determination of salt-sensitive HTN is illustrated in Figure 1.

Finally, because the evaluation of sodium intake through diet is subject to recall bias, it is crucial that investigators confirm sodium intake through other means. In that regard, verification of sodium intake from several 24-h urine collections is the gold standard approach. Other means of estimating sodium intake include spot urine measurements. This method eliminates the inconvenience and unfeasibility of 24-h urine collections but is subject to considerable variability (Elijovich et al., 2016).

## 2 Pathogenesis of salt-sensitive hypertension

### 2.1 Renin-angiotensin-aldosterone system dysfunction in salt-sensitive hypertension

A hallmark of salt-sensitivity is an overactive Renin-Angiotensin-Aldosterone System (RAAS), which has direct vasoconstrictor and anti-diuretic effects, leading to increased systemic vascular resistance (SVR) and BP (Varagic et al., 2008; Susic et al., 2009). Alterations in RAAS activity can be due to factors like age, race, sex, co-morbidities, and genetics (Elijovich et al., 2016).

Normally, low salt intake significantly increases RAAS components, including Angiotensin II (Ang II) and aldosterone, which promotes sodium reabsorption (Huan et al., 2012; Fujita, 2014). This is not the case in salt-sensitive humans, who exhibit reduced renin stimulation in response to salt depletion and inadequate suppression of renin after high salt intake (Kobori et al., 2003; Yatabe et al., 2010; Susic et al., 2011; Fujita, 2014). In other words, individuals with salt-sensitivity suffer from a bidirectional dampening of renin activity in response to variations in sodium consumption (Laffer et al., 2003; Laffer and Elijovich, 2013; Mishra et al., 2018). In addition to a systemic RAAS, several studies have examined the contribution of an independently functioning intrarenal renin-angiotensin system (RAS) in mediating progressive kidney disease in salt-sensitive HTN (Bayorh et al., 2005; Susic et al., 2011). For instance, Dahl salt-sensitive rats demonstrated elevated activation of intrarenal RAS after salt loading (Kobori et al., 2003). This was also observed in spontaneously hypertensive rats; RAAS/RAS activity was not suppressed and even amplified after exposure to a 4-week high salt diet (Susic et al., 2011). Moreover, treatment with the angiotensin receptor blocker (ARB) losartan ameliorated salt-induced renal injury by significantly reducing proteinuria, urinary angiotensinogen (AGT) excretion, glomerular injury, and interstitial fibrosis (Varagic et al., 2008; Susic et al., 2009; Susic et al., 2011).

Interestingly, rats treated with a chronic Ang II infusion in conditions of salt loading exhibit significant increases in kidney infiltration of immune cells, which augments renal reactive oxygen species (ROS) activity, tissue injury and urinary excretion of AGT, demonstrating an essential immunological component of RAAS-induced renal damage (Ozawa et al., 2007; Lara et al., 2012; Itani et al., 2016a; Norlander et al., 2017). In support of this, an increase in kidney immune cell infiltration has been observed across several salt-sensitivity models, including rodents treated with nitric oxide (NO) inhibitors, Ang II, or mineralocorticoids (Ozawa et al., 2007; Pechman et al., 2008; Itani et al., 2016a; Norlander et al., 2017). The immune system’s role in salt-sensitive HTN development will be discussed in more detail in Section 2.6.

### 2.2 Aldosterone-dependent and aldosterone-independent mineralocorticoid receptor signaling in salt-sensitive hypertension

Accumulating evidence has supported the role of aldosterone-dependent and aldosterone-independent mineralocorticoid receptor (MR) signaling in salt-sensitive HTN and organ damage (Farjah et al., 2003; Aoi et al., 2007; Shibata et al., 2011; Mishra et al., 2018; Bovée et al., 2021). Aldosterone activates MRs, which elevate BP through facilitating sodium reabsorption in the distal tubule and the collecting duct (Ayuzawa and Fujita, 2021a). Typically, high salt intake inhibits RAAS, which suppresses plasma aldosterone levels and MR activation, to maintain normal BP (Ishii et al., 1983; Kawarazaki and Fujita, 2013). However, salt-sensitive individuals demonstrate abnormally elevated MR signaling despite low aldosterone levels. This is because salt stimulates Rac1, a Rho family GTPase in salt-sensitive individuals, directly activating MR independently of aldosterone. By acting upstream of MR, Rac1 is a key regulator of BP response in salt-sensitivity through its on/off switching of MR activity (Mishra et al., 2018).

The mechanism behind aberrant activation of Rac1 is unclear. This may occur due to a genetic alteration or through activation by other stimuli (Deng et al., 2001; Palijan et al., 2003). In that regard, Rac1 stimulation has been observed in response to inflammatory cytokines (Papaharalambus et al., 2005), ang II (Schmitz et al., 2001; Nishida et al., 2005) and ROS (Miyata et al., 1998; Nagase et al., 2012; Hyndman et al., 2013), which are all upregulated in salt-sensitive HTN (Kirabo et al., 2014a; Kirabo et al., 2014b; Jin and Vaziri, 2014; Barbaro et al., 2017).

An important regulator downstream of MR is serum and glucocorticoid-regulated kinase 1 (SGK1), a known intracellular sensor of salt that is upregulated after salt loading (Fujita, 2014)*.* SGK1 activates the Na^+^-Cl^−^-Co-transporter (NCC) and Epithelial Sodium Channel (ENaC) in the kidney, augmenting renal sodium reabsorption and BP (Mishra et al., 2018). In this regard, the Dahl salt-sensitive rat, an important model of salt-sensitivity, demonstrated HTN and proteinuria after a salt load, which was associated with elevated Rac1 activation, MR signaling and upregulation of both SGK1 and ENaC, in comparison to Dahl salt-resistant rats (Farjah et al., 2003; Aoi et al., 2007; Shibata et al., 2011; Pavlov and Staruschenko, 2017). Treatment with either eplerenone, an MR antagonist, or NSC23766, a small molecule inhibitor of Rac1, ameliorated the salt-sensitive phenotype. Likewise, inhibition of ENaC using amiloride or benzamil significantly attenuated HTN development in Dahl salt-sensitive rats (Kakizoe et al., 2009; Pavlov et al., 2013), making ENaC an important therapeutic target for the treatment of salt-sensitive HTN. In addition to aldosterone, other factors such as the RAAS, arginine vasopressin (AVP), epidermal growth factor (EGF) and atrial natriuretic peptide (ANP) all regulate ENaC activity (Wang et al., 2006; Sun et al., 2011; Guo et al., 2013; Pavlov et al., 2013; Mironova et al., 2015; Rossier et al., 2015).

### 2.3 Sympathetic nervous system dysfunction in salt-sensitive hypertension

The sympathetic nervous system (SNS) is a key modulator of renal function and BP, and human and animal studies have demonstrated SNS overactivity in salt-sensitive HTN (DiBona, 2005; Ando and Fujita, 2012; Lohmeier et al., 2012). In response to a high salt diet, salt-sensitive hypertensive individuals exhibit higher levels of circulating norepinephrine than salt-resistant individuals, which indicates the persistence of a sympathetic drive after a salt load (Fujita et al., 1980; Campese et al., 1982; Gill et al., 1988)*.* In addition to this, salt-sensitive rats display compromised baroreceptor reflex control of sympathetic activity, indicating sodium-nervous system dysfunction through suppression of negative feedback mechanisms (Ono et al., 1994; Ono et al., 1997).

Renal denervation inhibits salt-induced BP elevation in patients with salt-sensitive HTN (Fujita and Sato, 1992; Krum et al., 2009; Esler et al., 2010), demonstrating an important role in kidney-specific sympatho-activation. In that regard, SNS overactivation induces renal anti-natriuresis in three ways: stimulation of renin secretion, reduced kidney perfusion, and increased tubular sodium reabsorption (DiBona, 2005). Regarding the third mechanism, evidence has demonstrated a direct effect of renal SNS activation on tubular sodium homeostasis and overall BP through modulation of NCC, a principal sodium transporter in the tubules (Lalioti et al., 2006). As such, two important pathways responsible for NCC activation have been proposed: the Rac1-MR-SGK1-NCC (discussed earlier) and the beta 2 adrenergic receptor (β2AR)- glucocorticoid receptor (GR)- With-no-lysine kinase 4 (WNK4)-NCC pathways. Sodium reabsorption occurs through aberrant renal β2AR-GR-WNK4 signaling, which activates NCCs in the distal convoluted tubule segment of the nephron (Fujita, 2014). WNK4 is an important inhibitor of NCC activity, and its activation results in renal sodium excretion (Yang et al., 2003; Zhou et al., 2012). In conditions of salt-sensitivity, SNS overstimulation activates renal β2ARs which in turn promote GR activation and subsequent WNK4 down-regulation and increased NCC activity (Mu et al., 2011; Fujita, 2014; Luzardo et al., 2015). Interestingly, epigenetic modulation is involved in WNK4 downregulation by GR (Mu et al., 2011). Activating the *β*2AR results in histone deacetylase-8 (HDAC8) inhibition, resulting in decreased transcriptional activity of WNK4 (Li et al., 2008).

### 2.4 Volume-loading theory of salt-sensitive hypertension: Renal mechanisms of salt-sensitive hypertension

There has been much controversy on the precise role of the kidney in initiating salt-sensitive HTN. Many have argued that a certain degree of kidney dysfunction and early sodium retention is necessary to initiate the series of events leading to salt-sensitivity (Coleman and Guyton, 1969; Guyton, 1992; Hall et al., 1999). Lending further evidence to this claim, many genetic alterations identified to date relate to natriuresis and strongly suggest that salt-sensitivity is principally dependent on faulty renal sodium handling. In this section, we will describe what is known as the “Volume-Loading” theory of salt-sensitivity (De Nicola et al., 2004; Morris et al., 2016; Borrelli et al., 2020).

Due to kidney injury, impaired natriuresis results in positive sodium and fluid balance, which rapidly causes ECV expansion. This leads to increased cardiac output (CO), largely through RAAS activation, in a maladaptive attempt to increase kidney perfusion pressure, preserve glomerular filtration rate (GFR), stimulate renal sodium and fluid excretion, and restore physiological plasma sodium concentrations (De Nicola et al., 2004; Borrelli et al., 2020). As BP is a product of CO and peripheral vascular resistance, salt-sensitive individuals experience a significant rise in BP in response to a salt load. In other words, the initiating driving force behind BP elevation is the early increase in ECV and CO. SVR is not increased until later, through hemodynamic autoregulation, to protect tissues from over-perfusion. Moreover, salt-sensitive individuals demonstrate diminished kidney feedback regulation, which further worsens the BP response (Koomans et al., 1982; Campese et al., 1991; Karlsen et al., 1997; Bidani et al., 2009; Burke et al., 2014; Ren et al., 2014).

This theory of salt-sensitivity is pioneered by the work of Guyton and others who demonstrated that the pressure–natriuresis curve of salt-sensitive hypertensive patients is shifted to the right and has a lower slope than that of their salt-resistant normotensive counterparts (Guyton, 1992; Hall et al., 1999). This indicates that salt-sensitive individuals require a higher BP to excrete the same amount of sodium. As such, Guyton’s hypothesis is built on the premise that the salt-sensitive phenotype involves impairment in systems that regulate adaptation to a salt load, placing the kidney at the center of fluid and sodium regulation with a quintessential goal of achieving a “zero balance” (Guyton, 1992; Olde Engberink et al., 2020). Recent observations questioned this conventional understanding and demonstrated that sodium could accumulate in hyperosmolar concentrations in tissues such as skin and muscle (Kopp et al., 2013), or the endothelium (Olde Engberink et al., 2015). More recent evidence indicates that excess salt intake can impair endothelial function and influence total peripheral resistance (DuPont et al., 2013; Jablonski et al., 2013). From this point of view, new concepts supporting a “vaso-dysfunction theory” have been put forth. This theory deems SVR and endothelial dysfunction as the major culprits in salt-sensitive pathogenesis. The “vaso-dysfunction theory” of salt-sensitivity will be discussed in the next section.

### 2.5 Vaso-dysfunction theory of salt-sensitive hypertension

In contrast to the conventional “Volume-Loading” theory of salt-sensitivity, which asserts that kidney damage is needed for the initiation of salt-sensitive HTN (Sullivan et al., 1987; Greene et al., 1990a; Khalil, 2006; Schmidlin et al., 2007a; Schmidlin et al., 2011), the “Vaso-Dysfunction” model argues that it is an impairment in vascular dilation that is the causative factor in that regard (Morris et al., 2016).

**FIGURE 2:**
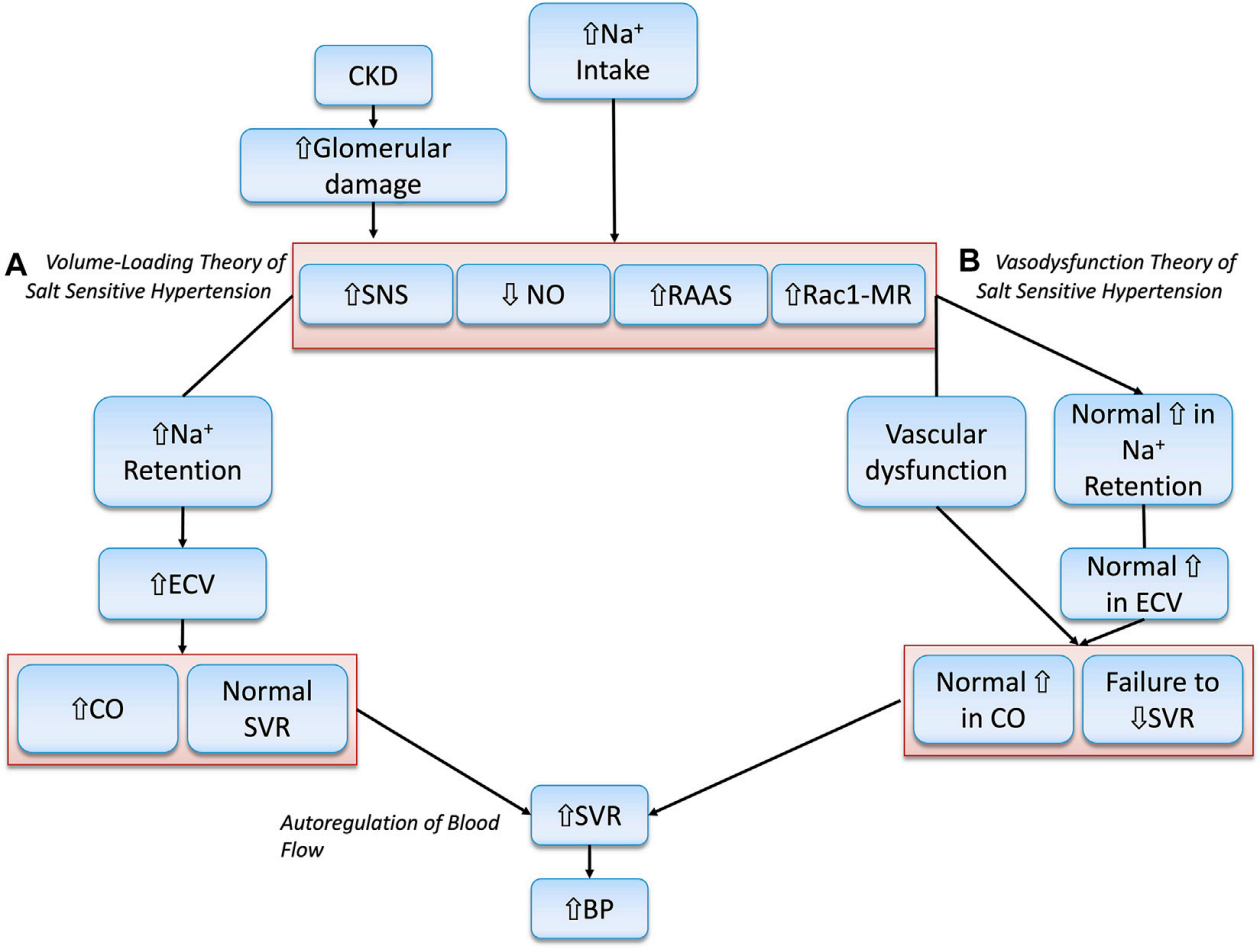
Illustration of the Volume-Loading” theory and the “Vaso-dysfunction” theories of Salt-Sensitive HTN. **(A)** Abnormally increased Na + retention, ECV and CO are the principal drivers of salt-sensitive HTN according to the “Volume-loading” theory. **(B)** This is in disagreement with the “Vaso-dysfunction” theory of salt-sensitive HTN that considers vascular dysfunction, or compromised ability to lower SVR, as the chief initiator of salt-sensitivity pathogenesis.

Proponents of this model maintain that salt-sensitive individuals do not retain larger amounts of sodium or exhibit larger increases in CO than their salt-resistant counterparts. However, only salt-resistant individuals can offset the hypertensive effect of increased CO by adequately decreasing SVR. In salt-sensitive individuals, failure to sufficiently decrease SVR, or even a paradoxical increase in vascular resistance, occurs from impaired vasodilation and is accompanied by a normal increase in CO. The subnormal decrease in SVR, without any aberrantly large increases in sodium retention or CO, is responsible for salt-induced BP elevation. This concept stemmed from studies that included salt-resistant control animals and/or human subjects who demonstrated an ability to vasodilate that starts within 12–24 h after a salt load, and that exceeds vasodilation in salt-sensitive patients (Ganguli et al., 1979; Sullivan et al., 1987; Simchon et al., 1989; Greene et al., 1990a; Schmidlin et al., 2007b; Schmidlin et al., 2011). This observation indicates that the impaired vascular resistance response is not a consequence of salt-induced increases in ECV and BP (Morris et al., 2016). Multiple mechanisms have been put forth to explain subnormal vasodilation in salt-sensitivity, including alterations in NO and SNS activity, aberrant inflammation, and increased vascular ROS (Bayorh et al., 2004; Morris et al., 2016). The “Volume-Loading” theory and the “Vaso-dysfunction” theory are shown below in Figure 2.

### 2.6 Immune systems mechanisms of salt-sensitive hypertension

**FIGURE 3:**
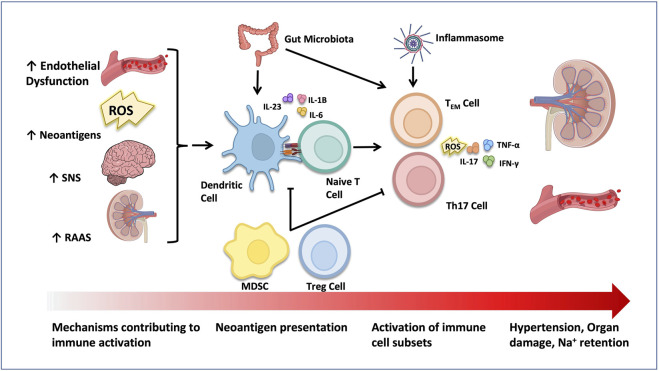
Activation of the immune system in HTN. Elevated SNS, RAAS and aldosterone activity, as well as increased ROS generation and neoantigen production have all been shown to activate immune cells in salt-sensitive HTN. Tregs and MDSCs play an anti-inflammatory role and suppress immune cell activation. The gut microbiome and inflammasome act on dendritic cells and/or T cells to contribute to HTN progression. Activated pro-inflammatory immune cells infiltrate target organs like the kidney and the vasculature and stimulate organ damage through production of ROS and pro-inflammatory cytokines.

Early evidence shed light on the important role of the immune system in salt-sensitive pathophysiology (Khraibi et al., 1984; Rodríguez-Iturbe et al., 2002a). In those studies, the administration of immunosuppressants like cyclophosphamide (Khraibi et al., 1984) or mofetil mycophenolate (MMF) (Rodríguez-Iturbe et al., 2002a; Pechman et al., 2008; De Miguel et al., 2010), decreased renal immune cell infiltration and oxidative stress and ameliorated HTN in rats. The role of immune system activation in salt-sensitive HTN is illustrated in Figure 3.

Later evidence suggested that Na^+^ can accumulate in tissues and promote inflammation (Machnik et al., 2009; Kopp et al., 2013; Titze, 2014). Specifically, salt can activate central components of both innate and adaptive immunity, such as the damage-associated molecular patterns (DAMPs), the complement system, the inflammasome, as well as dendritic cells (DC), T cells and macrophages (Harrison et al., 2011; Wenzel et al., 2011; Binger et al., 2015; Jantsch et al., 2015; Wenzel et al., 2016). In line with this, mice with severe combined immune deficiency (SCID) or recombination-activating gene 1 (RAG-1) gene deficiency (absence of B and T cells), demonstrated resistance to HTN, vascular abnormalities, renal damage, and water and salt retention, caused by Ang II or salt (Guzik et al., 2007; Crowley et al., 2010; Marvar et al., 2010; Mattson et al., 2013). On the other hand, adoptive transfer of T cells into mice with RAG-1 deficiency reinstates HTN and end-organ damage (Mattson et al., 2013). A pivotal study demonstrated how salt leads to the activation of DCs (Kirabo et al., 2014a). Na^+^ enters DCs through ENaC and is replaced with calcium (Ca^2+^) through the action of the Na^+^/Ca^2+^ exchanger. Ca^2+^ influx stimulates protein kinase C (PKC), which increases NADPH activity, driving the formation of isolevuglandins (isoLGs). These reactive lipid oxidation products are highly immunogenic when formed in DCs and can covalently interact with cellular proteins forming IsoLGs-proteins adducts. Activated DCs then release IL-6 and IL-1β and present isoLG-modified proteins to naïve T cells, leading to CD8^+^ and CD4^+^ T cell proliferation in secondary lymphoid organs (Kirabo et al., 2014a). In that regard, adoptive transfer of DCs cultured in high salt media to WT mice, promoted a hypertensive response to a sub-pressor dose of Ang II, an effect that was prevented by adding isoLG scavenger 2-HOBA, highlighting the role of DCs in salt-sensitive HTN. Importantly, activation of T cells requires the interaction of the antigen-presenting major histocompatibility complex (MHC) with the T-cell receptor (TCR), and of costimulatory molecules such as CD80 (B7-1) and CD86 (B7-2) on antigen-presenting cells (APC) which bind to CD28 on T cells (Vinh et al., 2010). Blockade of co-stimulation with an anti-CTLA4 immunoglobulin prevents HTN development, immune cell activation and vascular inflammation in Ang II- and DOCA/salt-treated mice. These effects were also observed in Ang-II treated mice lacking B7 ligands (Vinh et al., 2010). In that regard, significant increases of T cells and pro-inflammatory cytokines in the blood, perivascular tissue and kidney have been seen consistently in experimental animal models and humans with HTN (Olsen, 1972; Guzik et al., 2007; Mattson, 2010; Trott et al., 2014).

NaCl has also been shown to activate T cells directly, without help from APCs. For example, salt can drive the polarization of naive T cells into Th17 cells, a subset of CD4^+^ T helper cells, that produce interleukin-17A (IL-17A) which plays an important role in salt-sensitive HTN (Kleinewietfeld et al., 2013; Leavy, 2013). Interferon-γ (IFN-γ), another crucial proinflammatory cytokine secreted primarily by cytotoxic CD8^+^ T cells, also contributes significantly to salt-sensitive HTN (Garcia et al., 2012; Pang et al., 2012; Itani et al., 2016a). In the kidney, IL-17A and IFN-γ modulate the intrarenal production of Ang II and the activity of numerous renal sodium transporters, including the NCC and the sodium chloride co-transporter (NKCC) and sodium hydrogen exchanger-3 (NHE3) (Kamat et al., 2015a). They are also major players in vascular oxidative stress generation and aortic stiffening (Yan et al., 2008; Mir et al., 2009; Madhur et al., 2010a; Nguyen et al., 2013; Wu et al., 2014; Kamat et al., 2015b; Small et al., 2018), through the regulation of endothelial nitric oxide synthase activity (Yan et al., 2008; Nguyen et al., 2013). In that regard, mice lacking IL-17A are protected against BP, vascular dysfunction, renal sodium retention, and injury induced by Ang II (Madhur et al., 2010b; Norlander et al., 2016a; Norlander et al., 2016b). These mice demonstrated decreased renal NHE3 expression, albuminuria, aortic T cell infiltration, and ROS generation during HTN (Madhur et al., 2010b; Norlander et al., 2016a). Likewise, mice lacking IFNγ^−/−^ were also protected from HTN after chronic aldosterone (Garcia et al., 2012) or Ang II infusion (Trott et al., 2014; Kamat et al., 2015b) or during the high salt-feeding phase of N (gamma)-nitro-L-arginine methyl ester/High salt (L-NAME/HS) (Itani et al., 2016a). Moreover, less renal damage was seen as reflected by fibrosis, albuminuria, and relative neutrophil gelatinase-associated lipocalin (NGAL) expression, in mice lacking IFN-γ (Markó et al., 2012; Itani et al., 2016a).

#### 2.6.1 immunological memory in salt-sensitive hypertension

More recently, our group discovered a crucial role for immunological memory in the development of salt-sensitive HTN (52). Using two models of recurrent exposure to hypertensive stimuli, high dose Ang II infusion followed by low dose Ang II infusion or low dose L-NAME followed by HS exposure, the authors demonstrated the formation of HTN-specific effector memory T (T_EM_) cells. Once formed, these cells sensitize the host to subsequent, even mild hypertensive challenges that are not expected to increase BP on their own. This has been supported by other groups, who also detected T central memory (T_CM_) cells and T_EM_ cells in the blood, vasculature, and kidneys of hypertensive mice (Guzik et al., 2007; Matrougui et al., 2011). Importantly, T_EM_ cells can infiltrate the kidney and the vasculature and release inflammatory cytokines, promoting further Na^+^ retention, and direct kidney and vascular damage. When T_EM_ cells are transferred from the bone marrow of mice treated with L-NAME/HS to wild type mice, these cells homed to the bone marrow of wild type recipient mice and expanded with salt feeding, indicating that these cells are reactivated upon exposure to salt. Importantly, T_EM_ cells are also principal sources of IFN-γ and IL-17A in the kidney, significantly contributing to HTN and end-organ damage. In fact, mice lacking IFN-γ fail to develop HTN-specific memory T cells and kidney damage after exposure to L-NAME/HS (Itani et al., 2016a).

In addition to the above, a study by the same group examined the activation of human T cells in HTN, using a bone/liver/thymus (BLT) humanized mouse model (Itani et al., 2016b). This unique model employs mice with a functional human immune system, achieved through co-transplantation of human fetal thymus/liver tissues and hematopoietic stem cells in immunodeficient SCID mice (Lan et al., 2006). In response to Ang II, humanized mice demonstrated elevated BP and increased infiltration of CD4^+^ T cells in the thoracic lymph nodes, aorta, and kidney, as well as an increase in CD4^+^ and CD8^+^ memory T cells in the aorta and lymph nodes when compared to sham-treated controls. In humans, increased circulating CD4^+^ and CD8^+^ T cells were observed in peripheral blood samples obtained from hypertensive subjects. Intracellular staining for IFN-γ and tumor necrosis factor-α (TNF-α) demonstrated higher levels of IL-17A in CD4^+^ T cells and higher levels of both IFN-γ and TNF-α in CD4^+^ and CD8^+^ T cells, in hypertensive subjects.

#### 2.6.2 Inflammasome activation in salt-sensitive hypertension

Multiple vasoactive molecules, including endothelin-1, aldosterone, Ang II, and NaCl, trigger inflammasome activation in HTN (Caillon and Schiffrin, 2016). Specifically, the NLRP3 inflammasome has been shown to play a key role in the chronic inflammation that is seen in HTN (Dalekos et al., 1997; Rabkin, 2009). The first step of inflammasome activation is a priming phase where NF-*κ*B upregulates inflammasome components and pro-IL-1*β*. The second activation involves assembling the components into the NLRP3 inflammasome signaling complex. This leads to the formation of a multiprotein complex with a caspase-mediated proteolytic activity that activates pro-inflammatory cytokines IL-1β and IL-18.

Interestingly, some NLRP3 gene polymorphisms are associated with HTN (Omi et al., 2006; Xu et al., 2019). Inflammasome inhibition was shown to alleviate renal inflammation in HTN in several recent studies (Bai et al., 2017; Chen et al., 2017; Zhao et al., 2018). Deleting the nlrp3 gene in Mice deficient prevented Ang II-(169)and aldosterone-induced (Bai et al., 2017) podocyte injury. In a mouse deoxycorticosterone acetate (DOCA)-salt model, the NLRP3 inflammasome assembly inhibitor MCC950 attenuated HTN development and reduced collagen and pro-inflammatory gene expression in the kidney. Stemming from the above evidence, additional studies are needed to better understand the inflammasomes’ role, including non-NLRP3 inflammasomes, in HTN and salt-sensitivity.

#### 2.6.3 Myeloid-derived suppressor cells in salt-sensitive hypertension

The emergence of single-cell sequencing revolutionized the field of immunology, allowing for identifying unspecified immune cell populations. In that regard, a distinct subpopulation, peripheral CD11b^+^Gr1^+^ myeloid cells or myeloid-derived suppressor cells (MDSCs) accumulate in Ang- and L-NAME/HS- induced murine models of HTN (171). Depletion of MDSCs increased BP, renal T cell infiltration and cytokine expression. Interestingly, this population inhibited T cell activity through increased hydrogen peroxide production, demonstrating an important protective role for ROS in immunosuppression in HTN. Adoptive transfer of MDSCs reduced BP in hypertensive mice (Shah et al., 2015; Chiasson et al., 2018). A recent study demonstrated an immunosuppressive role for ubiquitin-editing protein A20 in DCs in HTN (Lu et al., 2019). Deletion of A20 resulted in increased BP, renal effector memory T cell infiltration and renal TNF-α and IFN-γ expression. The above studies demonstrate a cardioprotective role of myeloid cells subsets in HTN, emphasizing a self-regulatory role for the immune system in disease pathogenesis. The above studies demonstrate a cardioprotective role of myeloid cell subsets in HTN, emphasizing a self-regulatory role for the immune system in disease pathogenesis.

#### 2.6.4 Gut microbiota and salt-sensitive hypertension

Recent investigations have revealed an important role of the gut microbiota in developing HTN and other CVDs (Lau et al., 2017; Tang et al., 2017). The intestinal mucosa is rich in innate immune cells, which inspect the gut epithelia for antigens. Moreover, the intestine is the first and largest absorption site of Na^+^, making it an important potential site for immune cell activation (Elijovich et al., 2020). Short chain fatty acids (SCFA) are known metabolites of the intestinal microbiome which activate SCFA receptors such as FFA2 receptors on DCs and monocytes, leading to anti-inflammatory effects (Kim et al., 2014; Patrick et al., 2021). Important ligand agonists of the FFA2 receptors are 2-carbon acetate and the 3-carbon propionate which promote inhibition of NF-κB (Lee et al., 2013; Kim et al., 2014). The transfer of gut microbiota from hypertensive subjects (Li et al., 2017) or high salt fed mice (Ferguson et al., 2019), into germ-free mice increased BP in recipient mice. One important factor influencing the anti-inflammatory effect of the microbiota is gut microbial composition. Specifically, an increase in fecal Firmicutes/Bacteroidetes ratio is seen in hypertensive rats and humans (Yang et al., 2015), resulting from high salt intake in mice (Ferguson et al., 2019). In support of the above evidence, there are significant differences in microbiota composition between Dahl salt-sensitive and salt-resistant rats (Mell et al., 2015). Moreover, differences in microbiome composition among individuals may contribute to the interindividual variability in the BP responses to salt intake. Overall, more investigations are needed to better understand microbiome-induced immune activation mechanisms.

### 2.7 CKD and salt-sensitive hypertension

HTN is known to be both a leading cause and a consequence of chronic kidney disease (CKD) (Forouzanfar et al., 2017). According to the centers for disease control and prevention (CDC), the prevalence of HTN, defined as sustained BP ≥ 140/90 mmHg, in the US CKD population was 59.1% between 2013 and 2014 (Prevention CfDca, 2014). Several mechanisms contribute to HTN development in CKD, including RAAS and sympathetic system overactivity, Na^+^ and fluid retention, endothelial dysfunction, and immune activation. However, the cause-and-effect relationship between immunity, HTN and renal disease is only beginning to be understood.

Several salt-sensitivity models have observed an increase in immune cell infiltration, including rodents treated with NO inhibitors, Ang II, or mineralocorticoids (Ozawa et al., 2007; Pechman et al., 2008; Itani et al., 2016a; Norlander et al., 2017). The Dahl salt-sensitive rat, an important animal model of salt-sensitivity, shows progressive renal dysfunction through albuminuria and renal fibrosis when fed a high salt diet (De Miguel et al., 2010; Mattson, 2014). This is in line with the increased albuminuria seen in patients with salt-sensitive HTN (Bigazzi et al., 1994).

What mediates immune cell infiltration to the kidney? An interesting observation in Dahl salt-sensitive rats demonstrates that macrophages and T cells depend on an elevation of renal perfusion pressure to infiltrate the kidney (Mori et al., 2008). Damaged glomeruli and declining kidney function lead to increased activity of the RAAS in an attempt to preserve GFR. Elevated RAAS activity increases SVR, raising perfusion pressure and GFR of the remaining glomeruli. High Ang II levels and a net loss of overall GFR stimulate Na^+^ reabsorption in the proximal tubule and the collecting duct, further amplifying Na^+^ and fluid retention (Ku et al., 2019). The effect on BP is aggravated by increased salt-sensitivity (Koomans et al., 1982). A main characteristic of CKD is renal ischemia which leads to renal afferent nerve excitation through adenosine. This excitation, along with increased Ang II levels and Na^+^ retention, directly stimulated sympathetic outflow (Ku et al., 2019). Sympathetic system overactivation is exacerbated by compromised kidney autoregulation. Data obtained from individuals (Campese et al., 1991; Bidani et al., 2009; Burke et al., 2014) and experimental animals (Karlsen et al., 1997; Burke et al., 2014; Ren et al., 2014) with salt-sensitive HTN also demonstrated compromised renal vascular autoregulatory mechanisms.

Further supporting the role of the immune response in salt-sensitivity and kidney damage pathology, is the observation that renal immune cell infiltration occurs before an overt decline in kidney function. In that regard, obese Dahl salt-sensitive leptin receptor mutant (SS^LepR^mutant) rats as early as 4 weeks of age exhibit early development of renal injury that is associated with renal macrophage infiltration in comparison with lean salt-sensitive rats. Depletion of macrophages with clodronate for 4 weeks slows the early progression of renal injury SS^LepR^mutant rats through decreased glomerular injury and renal fibrosis (Poudel et al., 2020a). This is in line with other animal studies that observed early renal infiltration of macrophages that contributed to the development of HTN-induced kidney damage (Kitamoto et al., 2009; Thang et al., 2015; Huang et al., 2018; Poudel et al., 2020b).

A recent review elegantly described the influence of salt-sensitive HTN on distal tubular Na + reabsorption in the nephron. Aberrant Na + handling during CKD is due to multiple mechanisms that include aberrant aldosterone levels, intrarenal renin-angiotensin system (RAS) activity, high salt consumption, proteinuria and metabolic acidosis. Generally, a western diet produces a surplus of H+ which is excreted as ammonium. CKD patients exhibit an impaired ability to produce ammonia which compromises H+ neutralization and excretion (Moranne et al., 2009; Raphael et al., 2017). To restore acid-base homeostasis, the RAS and endothelin (ET) systems are activated to lower urine pH and promote H+ excretion. This is achieved through increased bicarbonate (HCO−3HCO3−) reabsorption and ammonium production in the proximal nephron (GeibelGiebisch and Boron, 1989; Levine et al., 1997; Nagami, 2002; Rothenberger et al., 2007; Wesson et al., 2012) as well as increased H + -ATPase activation in the distal tubules. Consequently, this alters the electrolyte gradient in the distal tubules, promoting Na + reabsorption. Proteinuria is another well characterized hallmark of CKD, which can be caused by podocyte injury. Nephrotic syndrome is a distinct set of kidney pathologies that cause substantial proteinuria and Na^+^ retention (Ahn and Bomback, 2020). A pivotal development in the understanding of nephrotic syndrome pathophysiology is the discovery that urine from nephrotic patients activates ENaC and independently increases Na^+^ retention (Svenningsen et al., 2009). This activation was caused by the proteolytic activity of urinary plasmin which increased the open probability of EnaC. Whether this mechanism extends to other forms of kidney disease or to salt-sensitive HTN remains unclear.

In addition to the above mechanisms, the role of amino acid metabolism in the pathophysiology of salt-sensitive HTN has gathered immense interest (Chen et al., 2016; Chen et al., 2019a; Feng et al., 2019; Rinschen et al., 2019; Rinschen et al., 2022). A crucial amino acid reported to play a central role in kidney disease progression in salt-sensitivity is lysine (Cheng et al., 2018; Rebholz et al., 2018; Chen et al., 2019b; Rinschen et al., 2022). According to a recent study, lysine supplementation in Dahl salt-sensitive rats attenuated HTN development and reduced kidney injury. The protective effects of lysine on the kidney were mediated through increased diuresis and excretion of central carbon metabolites as well as reduced tubular albumin uptake. Mechanistically, lysine conjugation to central carbon metabolites prohibits their modification of proteins and diuresis allows for the flushing out of protein remnants from the tubule system. This is consistent with human studies that detected alterations in lysine metabolism in hypertensive patients on protective low sodium diets (DASH) (Cheng et al., 2018; Rebholz et al., 2018; Chen et al., 2019b). Overall, high urinary lysine levels were associated with improved cardiovascular outcomes in HTN and CKD (McMahon et al., 2017; Cheng et al., 2018; Li et al., 2018; Rebholz et al., 2018; Chen et al., 2019b).

## 3 Sex-specific differences in salt-sensitive HTN

Large population studies have demonstrated higher rates of salt-sensitivity as well as an increased magnitude of salt-induced BP variation in women (Elliott et al., 1989; Bray et al., 2004; Chen, 2010; Blenck et al., 2016; Shukri et al., 2018; Faulkner and Belin de Chantemèle, 2020).

Evidence has shown that women, primarily those under the age of 51, exhibit a reduced ability to suppress aldosterone in response to stimuli such Ang II and salt intake (Faulkner et al., 2018; Shukri et al., 2018). Women typically exhibit higher aldosterone levels than men in several pathological states, such as salt-sensitive HTN (Shukri et al., 2018), primary aldosteronism (Ahmed et al., 2011), and obesity (Goodfriend et al., 1999), and appear to be more sensitive to endothelial damage (Safar et al., 2013; Huby et al., 2015; Huby et al., 2016). Two reports explained this observation by demonstrating the influence of sex hormones on adrenal physiology (Caroccia et al., 2014; Grabek et al., 2019). First, Caroccia et al. demonstrated a dimorphic receptor-dependent effect of estradiol on aldosterone synthesis (Caroccia et al., 2014). In another study, testosterone demonstrated an important inhibitory effect on adrenal gland tissue renewal (Grabek et al., 2019). Put together, it is possible that a high estrogen/testosterone ratio, as seen in women, is an important mediator of salt-sensitivity through differential aldosterone secretion.

In addition to elevated aldosterone levels, several studies point to MR activation and upregulation as being a principal player in development of salt-sensitive HTN in females (Faulkner et al., 2019). Endothelium-specific MRs are central mediators of endothelial dysfunction (Deanfield et al., 2007; Faulkner and Belin de Chantemèle, 2020). A recent study indicated that women and female mice express higher levels of MRs in the vascular endothelium and that this expression was driven by progesterone receptor activation (Faulkner et al., 2019). The same group then demonstrated that under baseline conditions, female Balb/C mice demonstrate greater vascular relaxation to acetylcholine and reduced constriction to phenylephrine, in comparison to their male counterparts. However, when these mice with put under a sodium-restricted diet to induce aldosterone levels, isolated aortic rings from female mice exhibited impaired relaxation and enhanced constriction responses, which abolished this baseline advantage (Faulkner et al., 2021). This supports earlier data from the group which demonstrated that sodium restriction significantly increased aldosterone levels only in female mice and deletion of the endothelial MR prevented female mice from developing endothelial dysfunction on salt restriction (Faulkner et al., 2020). Interestingly, sodium restriction also significantly reduced eNOS expression in female mice promoting endothelial impairment, and inhibition of eNOS with L-NAME also eliminated the difference in aortic relaxation between female mice on a normal-salt diet and those on a salt-restricted diet (Faulkner et al., 2021). Put together, it is possible that higher aldosterone levels combined with upregulation of endothelial MRs, increases susceptibility to endothelial MR activation in salt-sensitive females, predisposing them to aldosterone-dependent NO-mediated endothelial dysfunction that contributes to sex specific HTN and cardiovascular risk.

In support of the above experimental data and clinical trials found a more significant reduction in BP and cardiovascular risk upon treatment with MR antagonists in females than in males (Olivieri et al., 2008; Kanashiro-Takeuchi et al., 2009; Gwoo et al., 2014; Faulkner et al., 2018).

## 4 Animal models of salt-sensitive hypertension

The Dahl salt-sensitive rat is a very useful example for studying salt-sensitive HTN. When fed with high diets, these rats characteristically develop HTN and proteinuria (Dahl et al., 1962; Dahl et al., 1963; Khan et al., 2012). The increase in BP is attributable to sodium retention in this model and is abrogated upon treatment with diuretics (Tobian et al., 1979; Greene et al., 1990b). In addition, the genetic analysis demonstrated important influences of alleles at the angiotensin-converting enzyme (ACE) and guanylyl cyclase A (GCA)/atrial natriuretic factor (ANF) receptor gene loci on BP in these animals (Deng and Rapp, 1992). Dahl salt-sensitive rats also demonstrate endothelial dysfunction, glomerulosclerosis and cardiac hypertrophy, cardiac fibrosis (Raij et al., 1984; Hayakawa et al., 1997; Yu et al., 2003), as well as T cell and macrophage infiltration in the cortex and medulla (Hayakawa et al., 1997; Mattson et al., 2006; De Miguel et al., 2010). Recently, NADPH oxidase 4 (NOX4) has emerged as a principal generator of ROS in this salt-sensitivity model. Renal NOX4 was shown to regulate ENaC activity in Dahl salt-sensitive rats, as genetic deletion of NOX4 prevented high-salt-induced increases in ENaC activity in the kidney as well as hydrogen peroxide (H_2_O_2_) generation, salt-induced BP elevation, albuminuria, tubular cast formation, and glomerular injury (Cowley et al., 2016; Pavlov et al., 2020). Another recent study further demonstrated an important NOX4/H_2_O_2_/mTORC1 pathway that contributes to salt-induced HTN and renal injury through immunomodulation (Kumar et al., 2020).

Another important model to study the mechanisms that govern salt-sensitive HTN, is the L-NAME/HS hypertensive mouse model. The treatment protocol involves adding a low dose (0.5 mg/ml) of L-NAME in the drinking water for 2 weeks, followed by a 2-weeks washout phase and a high salt diet (4%). An important advantage of this model is that it induces immunological memory (Itani et al., 2016a) through repeated hypertensive stimuli and without any surgical intervention. It mimics salt-sensitive HTN encountered in humans, making it a particularly useful tool for studying inflammation. L-NAME is an important nitric oxide synthase inhibitor, and inhibition of NO triggers events that induce endothelial dysfunction and stimulate the inflammatory response, both of which are key to developing salt-sensitivity (Van Beusecum et al., 2019). The ROS generated by NO inhibition increases the endothelial expression of adhesion molecules and chemokines (Le Brocq et al., 2008). This leads to monocyte transmigration and activation of myeloid-derived DCs, which then activate T cells and prime HTN development (Randolph et al., 1998; Kirabo et al., 2014b). Moreover, NO prevents lipid peroxidation and inhibits the formation of lipid peroxidation products like isoprostanes and isoketals, which are immunogenic in DCs. Another important pathogenic event resulting from low-dose L-NAME is vascular dysfunction (Wang et al., 2020). In that regard, rats treated with a low dose of L-NAME demonstrated Subnormal vasodilation and elevated BP in response to a salt load (Wang et al., 2020). Interestingly, in that study, volume expansion was comparable between the two groups on day 1, suggesting that vascular dysfunction was responsible for the initiation of salt-sensitive HTN. After day 1, volume expansion in salt-sensitive mice was associated with increased NCC, suggesting that volume expansion and sodium retention were necessary to maintain HTN. Importantly, the impairment of renal sodium handling and increased NCC activity seen in this model were consequences of ROS, not secondary to renal parenchymal fibrosis. Individuals with salt-sensitive HTN have been reported to generate less NO than their salt-resistant hypertensive counterparts (Ghiadoni et al., 1997), and many genetic variations modulating NOS activity are common in groups with a high prevalence of salt-sensitive HTN (Svetkey et al., 1996; Sverdlov et al., 2014; Elijovich et al., 2016).

The DOCA-salt-induced model has been widely employed to study salt-sensitive HTN. It involves the administration of DOCA in combination with surgical reduction of renal mass or unilateral nephrectomy and a high salt diet. Removal of kidney mass decreases renin production and fluid excretion, producing a volume overload and low renin form of HTN (Chamorro et al., 2004; Sun and Zhang, 2005; Leong et al., 2015). This increases CO and cardiac mass due to volume expansion, and is accompanied by proteinuria, glomerulosclerosis and endothelial dysfunction (Klanke et al., 2008; Iyer et al., 2010). Further studies have shown this model’s increased SNS and RAAS activity (Sawamura and Nakada, 1996).

In light of the above, recent study demonstrated the first spontaneous mouse model of sex-specific salt-sensitivity (Faulkner et al., 2018). Most experimental data to date has been obtained from male mice, and it has been revealed that the pathophysiological mechanisms underlying this type of HTN differ between males and females. JL Faulkner et al. demonstrated that female Balb/C mice develop salt-induced elevations in BP upon exposure to a 7-days high-salt diet, an effect that was not observed in male Balb/C mice. Moreover, female Balb/C mice on a high salt diet demonstrated reduced RAAS activity but exhibit higher levels of aldosterone synthase and aldosterone in comparison to males. Importantly high salt exposure resulted in impaired endothelium-dependent vasodilation in female mice only and treatment with eplerenone, a MR inhibitor decreased BP and improved endothelial function females (Faulkner et al., 2018). The above model demonstrated a enhanced sensitivity to aldosterone and MR activation that is strongly analogous salt-sensitive pathology in women (Safar et al., 2013; Huby et al., 2015; Huby et al., 2016; Shukri et al., 2018; Faulkner et al., 2019) (Refer to Section 3. “Sex-Specific Differences in Salt-Sensitive HTN”).

Genetically induced models of HTN involve animals with artificial genetic modifications that make them predestined to develop HTN. The mouse model for Liddle’s Syndrome is another useful genetic animal model for understanding the molecular and pathophysiologic events that lead to salt-sensitive HTN. Liddle’s syndrome is a monogenic form of salt-sensitive HTN, that is associated with hypokalemic metabolic alkalosis, suppressed plasma renin activity, low plasma aldosterone levels, and increased Na^+^ absorption in the kidney (Warnock, 1998). It results from an autosomal dominant mutation in the SCNN1B and SCNN1G genes, which code for the β and γ subunits of ENaC. These gain of function mutations lead to increased channel activity and elevated Na^+^ reabsorption in the distal nephron. Like the human disease, the mouse model generated by Pradervand et al. expresses a gain-of-function mutation in the β subunit of ENaC (Pradervand et al., 1999). Also similar to the human phenotype, this animal model is known to be normotensive until exposed to high salt, which induces HTN, hypokalemia, metabolic alkalosis, and cardiac and renal hypertrophy. The high sodium reabsorption and low plasma aldosterone levels under normal-salt diet indicate chronic hypovolemia, which is also typical of the human syndrome. Although this model simulates a rare and monogenic form of HTN, it remains a valuable research tool as, in essence, many of the genes and pathways involved in its pathogenesis would be obvious candidates for more common mutations that induce less extreme changes in BP. Identifying genes is crucial to developing personalized treatment strategies and precision medicine (30).

More recently, consomic or congenic strains on the Dahl salt-sensitive background are being generated to understand genetic contributions to salt-sensitive HTN pathophysiology (Kunert et al., 2006; Liang et al., 2008; Moreno et al., 2011a; Cowley and Dwinell, 2020). Examples include the consomic salt-sensitive-13^BN^ denoted as SS-13^BN^ or the salt-sensitive-18^BN^ denoted as SS-18^BN^, in which the Brown Norway (BN) chromosomes 13 or 18 are introduced into the Dahl salt-sensitive rat genome (Kunert et al., 2006). In comparison to the Dahl salt-sensitive rat, the SS-13^BN^ and SS-18^BN^ strains demonstrate important changes in vascular reactivity and are protected from salt-induced HTN development and albuminuria (Kunert et al., 2006). Chromosome 13 is a one of interest in HTN research as it contains the renin gene, which has been shown to carry a polymorphism in Dahl salt-sensitive rats (Cowley and Dwinell, 2020). Although consomic strains help link chromosomes to pathophysiological phenomena and disease, it is important to note that removal of a native chromosome will inevitably have other implications on organism function and disease, due to the possibility of complex genetic influences like gene-gene interaction, genetic heterogeneity, and gene penetrance as well as environmental factors.

In addition to consomic strains, further explorations led to the development of 26 congenic strains covering the entire length of chromosome 13 (Moreno et al., 2007). Authors identified 4 regions of chromosome 13 that independently afforded protection from salt-induced HTN development. Overall, these strains comprise a powerful tool to study allelic and genetic association with complex disease. They enable identification of a functional outcome of an allele or gene of interest against a fixed genetic background and with considerable percentage homology to the parental or control strain (Cowley and Dwinell, 2020). The discussed animal models above are summarized in Table 1.

**TABLE. 1:** Animal models used to study salt-sensitive HTN and hypertensive end-organ damage.

Animal models of salt-sensitive HTN	Genetically induced model of HTN	Advantages as a research tool	References
LNAME/HS hypertensive mouse model	No	Mimics salt-sensitive HTN encountered in humans	(Itani et al., 2016a)
		Induces immunological memory through repeated hypertensive stimuli and without any surgical intervention	(Itani et al., 2016a)
		Triggers endothelial dysfunction and inflammatory response	(Randolph et al., 1998; Van Beusecum et al., 2019; Wang et al., 2020)
DOCA-salt-induced hypertensive model	No	Characterized by to increase in CO and cardiac mass due to volume expansion, proteinuria, glomerulosclerosis and endothelial dysfunction	(Klanke et al., 2008; Iyer et al., 2010)
		Increases SNS and the RAAS activity	(Sawamura and Nakada, 1996)
Dahl salt-sensitive rat model	No	Induces HTN by increasing sodium retention and proteinuria when fed with high salt diets	(Dahl et al., 1962; Dahl et al., 1963; Khan et al., 2012)
		Promotes endothelial dysfunction, glomerulosclerosis and cardiac hypertrophy and fibrosis	(Raij et al., 1984; Hayakawa et al., 1997; Yu et al., 2003)
		Triggers T cell and macrophage infiltration in the cortex and medulla	(Hayakawa et al., 1997; Mattson et al., 2006; De Miguel et al., 2010)
Liddle’s Syndrome mouse model	Yes	Induces HTN, hypokalemia, metabolic alkalosis and cardiac and renal hypertrophy	(Warnock, 1998)
Consomic/Congenic/Subcongenic strains	Yes	Enables understanding of functional significance of a chromosome/gene/allele to disease pathophysiology	(Kunert et al., 2006; Liang et al., 2008; Moreno et al., 2011a; Cowley and Dwinell, 2020)
Female Balb/C mice	No	Exhibit higher levels of aldosterone synthase and aldosterone compared to males	Faulkner et al. (2018)
		Impaired endothelium-dependent vasodilation	
		Important for studying sex-specific differences in salt-sensitive HTN	

## 5 Treatment of salt-sensitive HTN

At the present time, there are no standardized therapeutic guidelines to treat salt-sensitive HTN. The consensus is that a reduction in salt intake at a population level combined with conventional antihypertensive therapy is the most effective option for HTN management in this subgroup (Mishra et al., 2018). The WHO classified sodium restriction as one of the most cost-effective methods to lower the burden of HTN and CVD. In that regard, evidence has strongly suggested the benefit of lowering sodium intake in both hypertensive and normotensive populations (Elliott et al., 1996; Sacks et al., 2001). The WHO recommends an upper limit of 2 g/day of salt consumption. However, most countries worldwide consume more than twice the daily limit, and compliance to dietary regimens remains low, mandating alternative approaches such as antihypertensive medications (Mishra et al., 2018).

The benefit of increasing dietary potassium has also gained interest (McDonough et al., 2017; Staruschenko, 2018; McDonough and Fenton, 2022). The Dietary Approaches to Stop Hypertension (DASH) diet, a US-based multi-center RCT (Akita et al., 2003), along with other studies, demonstrated that elevated potassium (K^+^) intake reduces BP in adults with HTN and is associated with a lower risk of death and cardiovascular events (O'Donnell et al., 2014; Terker et al., 2015; Pechère-Bertschi and Burnier, 2004). A potential downfall of increased K^+^ consumption is that high plasma K^+^ stimulates aldosterone secretion (Sorensen et al., 2013). In that regard, a “U-shaped” association between K^+^ consumption and BP was observed in a large meta-analysis of RCTs. According to that study, dietary K^+^ intake below 30 or above 80 mmol/day was associated with elevated BP (Filippini et al., 2017). In addition to regulation of electrolyte balance, enhanced K^+^ levels promote endothelium-dependent vasodilation through hyperpolarization K_ir_ channels, or “inward-rectifier” channels, which mediate large inward currents depending on extracellular K^+^ concentrations (Staruschenko, 2018). In that regard, the WHO recommends a potassium intake of at least 3,510 mg/d, to generate Na: K^+^ intake ratio of ≤0.6 mg/mg (WHO, 2012). However, global consumption reflects much higher ratios. Overall, further investigations are needed to understand the implications of K^+^ supplementation on salt-induced HTN.

Currently, many classes of antihypertensive medications are approved for HTN management, including diuretics, beta-receptor blockers, angiotensin-converting–enzyme inhibitors (ACEI), calcium-channel blockers (CCB), and ARBs (Mishra et al., 2018). A recent meta-analysis compared the efficacy of the above five classes of hypertensive drugs in salt-sensitive HTN. Researchers found that a combination of a CCB with hydrochlorothiazide, a diuretic, worked best for patients with moderate salt intake. For obese patients with moderate salt intake, a combination of a CCB with metformin was the most effective at lowering BP (Qi et al., 2018). For this review, we will discuss the more recent studies examining treatment options for salt-sensitive HTN.

In the United States, an estimated 43.7% of hypertensive individuals have their BP under control, or 38.9% when considering the cutoffs specified by the new AHA/ACC guideline (Muntner et al., 1999-2000). The global control rates are much lower at 21% (World Health Organization), 2021). Resistant HTN exists in a subgroup of hypertensive patients and is defined as uncontrolled BP despite adding three antihypertensive agents of different classes, including a diuretic. A more severe type of HTN is refractory HTN, where uncontrolled BP persists after 6 months of treatment with six or more antihypertensive agents (Acelajado et al., 2019). Importantly, both these types of HTN are more common among the black population, who also have a higher predisposition for salt-sensitive HTN. It also follows that the lack of adequate BP control indicates the involvement of other unaddressed mechanisms. In support of this, PATHWAY-2 sub study findings suggest that excess fluid retention mediated by high aldosterone levels are characteristic of resistant HTN (Pimenta et al., 2009; Williams et al., 2018).

In that regard, spironolactone, was shown to be a valuable add-on agent for the management of resistant HTN (284–286). Intriguingly, the BP lowering effects of spironolactone were less pronounced, although still useful, in those with refractory HTN (Acelajado et al., 2012); this supports the presence of a mineralocorticoid-independent stimulation of ENaC activity, which is typical of salt-sensitive HTN. Supporting this, a study in black hypertensive individuals demonstrated higher BP lowering responses with amiloride, an ENaC inhibitor, than spironolactone (Saha et al., 2005). Interestingly, amiloride was recently shown to inhibit the urinary serine protease plasmin, decreasing EnaC activation and Na^+^ retention in nephrotic mice (Bohnert et al., 2018) and in patients with resistant HTN and type 2 diabetes mellitus (Oxlund et al., 2014). Therefore, a future area of focus in salt-sensitivity research would be to delineate the effects of amiloride on renal plasmin activity in salt-sensitive HTN.

Recently, the Canakinumab Anti-Inflammatory Thrombosis Outcome Study (CANTOS) revealed an important role of the inflammasome in cardiovascular diseases (Ridker et al., 2017). Canakinumab, an anti-IL-1β antibody, significantly reduced cardiovascular events, including nonfatal myocardial infarction, nonfatal stroke, or cardiovascular death. While Canakinumab therapy did not result in a significant decrease in subjects’ BP, additional analysis revealed a trend toward a higher reduction in major adverse cardiac events in participants in the highest BP quartile (Thompson and Nidorf, 2018). To that end, additional investigations are crucial to improve the current understanding of the role of inflammasomes and their potential as therapeutic targets in salt-sensitive HTN.

Evidence has also suggested the possible therapeutic efficacy of azilsartan, a newly approved ARB. In salt-sensitive hypertensive mice, azilsartan improved salt-sensitivity by increasing the urinary excretion of sodium and the slope of the pressure-natriuresis curve. The underlying mechanism was a selective reduction in renal proximal tubule Na^+^/H^+^ exchange expression (Hatanaka et al., 2016). However, the therapeutic benefit of azilsartan does not appear to extend to all ARBs and ACEIs, as the pressure-lowering effects of enalapril, captopril and losartan were negated with high salt intake in human and animal studies (Xu and Brooks, 1997; HERLITZ et al., 1998; Fang et al., 1999).

ANP, an important player in the development of salt-sensitive HTN, is a hormone known to activate natriuresis, vasodilation and BP decline. Salt-sensitive hypertensive individuals exhibit suppression of ANP (Campese et al., 1996; Daniels et al., 2012). In that regard, an important recent study examined the effects of sacubitril, a neprilysin inhibitor, in Dahl salt-sensitive rats. Neprilysin is a metalloprotease that degrades ANP. The authors examined four treatment modalities: a combination of sacubitril and valsartan (75 μg/day each), sacubitril alone or valsartan alone or vehicle on renal function in Dahl salt-sensitive rats on a high salt diet for 21 days (Polina et al., 2020). Effects were classified as being sacubitril- or valsartan- or combination therapy-driven. As such, systolic BP decrease was primarily driven by valsartan as it was only seen in valsartan-treated groups. On the other hand, preservation of GFR was primarily sacubitril-driven as it was only seen in groups with sacubitril on board. Moreover, the three treatment groups demonstrated a significant reduction in urinary neutrophil gelatinase-associated lipocalin levels, but only valsartan-treated mice exhibited lower urinary KIM-1 excretion, indicating that alleviation of renal tubular damage may be primarily driven by valsartan. Finally, proteinuria and renal medullary fibrosis reduction appeared to be driven by combination therapy. To that end, 3 months of combination sacubitril/valsartan therapy was shown to be effective in alleviating kidney injury in mice with diabetic kidney disease (Myakala et al., 2021). Mechanistically, sacubitril/valsartan treatment exerts anti-oxidative and anti-inflammatory effects, through reduced NOX4 mRNA levels in db/db kidneys and reduced monocyte chemotactic protein-1 (MCP-1), Toll-like receptor 2 (TLR2), Cyclic GMP–AMP synthase (cGAS), and stimulator of IFN genes (STING) mRNA levels in KKAy mice.

The potential use of Na^+^-glucose cotransporter-2 inhibitors (SGLT2i) in salt-sensitive HTN and associated renal diseases has also generated much interest. A recent study found that dapagliflozin 2 mg/kg/day ameliorated HTN, improved pressure natriuresis and increased urinary flow rate, glucosuria, and Na^+^- and Cl^−^-to-creatinine ratios in Dahl salt-sensitive rats (Kravtsova et al., 2022). Interestingly, in that study, no changes in the expression of RAAS metabolites or in Na^+^ channels SGLT2, NKCC, NHE3, NCC and ENaC was observed. In another study, dapagliflozin 0.5 mg/kg per day decreased BP and sympathetic activity in prehypertensive spontaneously hypertensive mice as young as 8 weeks of age, at rest and during exercise, and prolonged treatment with dapagliflozin until 17 weeks of age promoted a sustained reduction in BP and HR which prevented HTN progression and resulted in lower heart weight (Kim et al., 2022). The favorable effect of dapagliflozin in conditions of exercise was not associated with any changes in blood glucose or plasma insulin or baroreflex function, indicating that dapagliflozin mediates a BP lowering response through sympathoinhibition. This is in line with other studies demonstrating lower renal and cardiac norepinephrine levels upon treatment with an SGLT2i in mice on a high-fat diet (Herat et al., 2020). Moving forward, randomized clinical trials are needed to determine efficacy and safety of SGLT2is in prevention and progression of salt-sensitive HTN.

## 6 Genetic background of salt-sensitive hypertension

**FIGURE 4:**
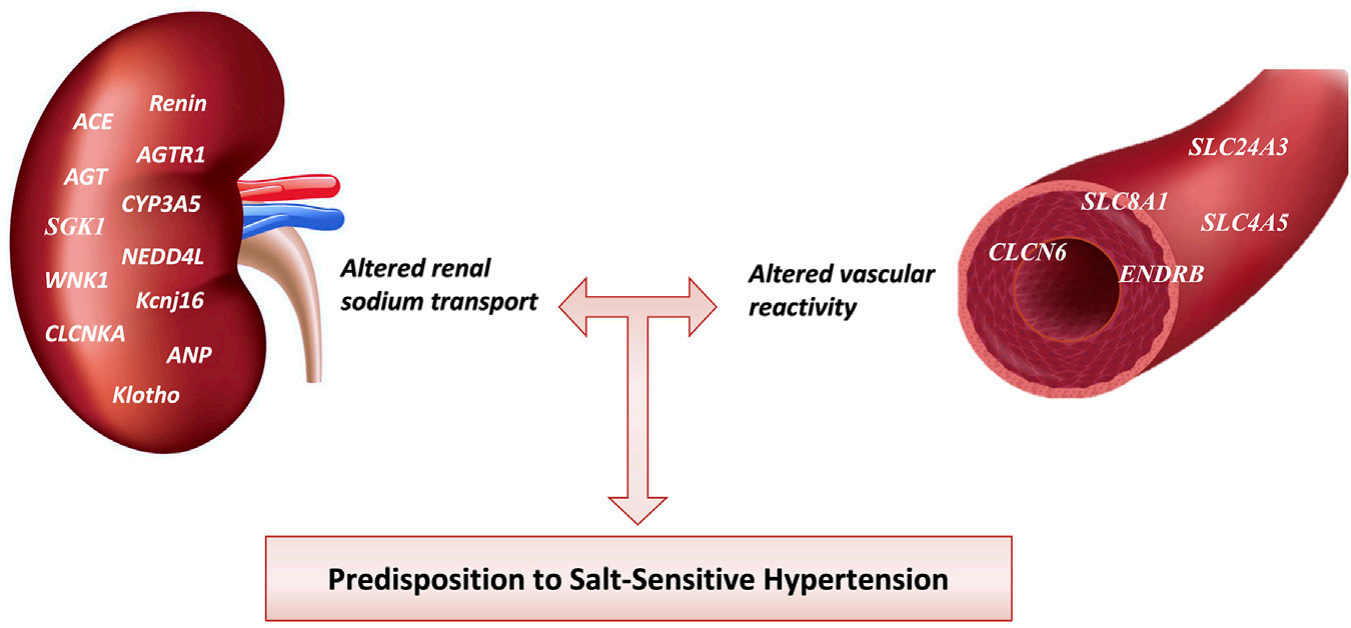
Genes involved in Salt-sensitive HTN pathophysiology. Different genetic mutations affect renal sodium transport and blood vessel reactivity increasing salt-sensitive HTN predisposition among individuals. ACE, angiotensin-converting enzyme; AGTR1, angiotensin receptor type 1; AGT, angiotensinogen; CYP3A5, cytochrome P-450 3A5; SGK1, serum and glucocorticoid-regulated kinase 1; NEDD4L, Neural precursor cell expressed developmentally downregulated gene 4-like; WNK1, With-no-lysine (K)-1; Kcnj16, Potassium inwardly-rectifying channel, subfamily J, member 16; CLCNKA, Chloride Voltage-Gated Channel Ka; ANP, Atrial Natriuretic Peptide; SLC24A3, solute carrier family 24 member 3; SLC8A1, solute carrier family 8 member 1; SLC4A5, solute carrier family 4 member 5; ENDRB, endothelin receptor type B; CLCN6, Chloride Voltage-Gated Channel 6.

Individual BP responses to minimal changes in dietary salt intake are heterogeneous. This may be linked to a genetic predisposition to HTN. The discovery of common allelic variations of candidate genes for HTN in relation to the salt-sensitive phenotype has been the focus of research. Genome-wide association studies have identified rare genomic variants influencing 26% of the population’s BP variability (Lip and Padmanabhan, 2020). Although such studies did discover some genes that modulate BP, candidate gene association analyses have identified genes linked to salt-sensitive HTN (305) (Refer to Table 2.). Since the kidney is a vital organ for long-term BP regulation, many studies have investigated the genomics of sodium handling anomalies in the renal tubules of salt-sensitive hypertensive individuals (Strazzullo and Galletti, 2007). On the contrary, a growing school of thought claims that aberrations of the genes that regulate vascular reactivity are involved in the pathogenesis of salt-sensitive HTN (Armando et al., 2015). The genetics of salt-sensitive HTN is shown in Figure 4.

**TABLE 2:** Allelic variants of candidate genes for HTN in relation to the salt-sensitive phenotype affect renal sodium transport or vascular reactivity.

	Candidate gene	Associated variants	Mode of action of the encoded protein	References
Genes of the Renin-angiotensin-Aldosterone system	Angiotensin-converting enzyme (ACE)	Insertion/Insertion and Insertion/Deletion genotypes	**Increase renal sodium transport**	Giner et al. (2000)
	Angiotensin II type 1 receptor	rs4524238		Iwai et al. (2007)
	Angiotensinogen (AGT)	M235T (rs699)		ElAlfy et al. (2019)
Genes related to Mineralocorticoids and aldosterone	11-B-Hydroxysteroid Dehydrogenase gene (11βHSD)	(rs45598932 G-209A)		Alikhani‐Koupaei et al. (2007)
	Serum- & Glucocorticoid-Regulated Kinase (SGK1)	rs2758151 and rs9402571		Rao et al. (2013)
	CYP3A5 gene	CYP3A5 *1 allele		Eap et al. (2007)
Genes related to renal ion transport	Neuronal precursor cells expressed developmentally down-regulated 4-like (NEDD4L)	rs4149601 GG-genotype together with the rs2288774 CC-genotype		(Manunta et al., 2008; Rizzo and Staub, 2015)
	Protein kinase, lysine-deficient 1 (WNK1)	rs880054		Manunta et al. (2008)
	Chloride channel, Kidney A (CLCNKA)	rs5718 (G-173A)		Barlassina et al. (2007)
Atrial Natriuretic peptide	ANP gene	Homozygous deletion		Mishra et al. (2018)
Klotho gene	Klotho gene (KL)	rs9536314		Citterio et al. (2020)
Sodium bicarbonate exchangers	Solute Carrier Family 24 Member 3 (SLC24A3)		**Increase vascular reactivity**	(Citterio et al., 2011; Yang et al., 2017)
	Solute Carrier Family 8 Member 1 (SLC8A1)	rs7571842 and rs10177833		(Blaustein et al., 2009; Carey et al., 2012)
Endothelin (ET) system	Endothelin Receptor type B gene (ENDRB)	rs5351		Caprioli et al. (2008)

### 6.1 Genes related to increased renal sodium transport

HTN can be caused by a number of single-gene mutations that directly impact renal salt reabsorption, however, these variants have only been seen in a few people. So far, phenotypic investigations have had difficulty distinguishing salt-sensitive from salt-resistant people. Thus, researchers are increasingly focusing on discovering some genes that may be implicated in salt-sensitivity. This section sheds light on the genetic variants in the renal system that appear to be of particular relevance in salt-sensitive HTN pathogenesis.

#### 6.1.1 genes of the renin-angiotensin aldosterone system

The RAAS is central in maintaining BP homeostasis by regulating renal sodium transport. The interplay of numerous RAAS components expressed in cells from various organs can alter crucial physiological properties such as cardiac excitability and ionic channel activation in order to maintain consistent BP (De Mello and Frohlich, 2014). Reasonably, genetic variations in genes involved in the renin-angiotensin system predispose salt-sensitivity in carriers (Sanada et al., 2011). To start with, renin is one of the primary players in the activation of the RAAS chain reactions, and it acts to cleave angiotensinogen into angiotensin I. The first renin knockout (Ren^−/−^) rat with salt-sensitive HTN background was developed in 2011 using the zinc-finger nucleases (ZFN) technology. ZFN designed to target the renin gene, causing a 10-bp deletion and resulting in a frameshift mutation. It was observed that Ren^−/−^ rats experienced a reduction of about 50 mmHg in their blood pressure in comparison to Ren^+/-^ littermates, when on low-salt diet (0.4% NaCl) (Moreno et al., 2011b). In their pivotal work, Moreno and colleges suggested that the decrease in basal blood pressure of renin knockout rats was due to an attenuation in the steroidogenesis process occurring in the zona glomerulosa of the adrenal cortex (Raff et al., 2015). Furthermore, the expression and activity of renal sodium channels and transporters were significantly dysregulated in renin-deficient salt-sensitive rats (Pavlov et al., 2016). Some polymorphisms associated with salt-sensitive HTN include polymorphism in the angiotensin-converting enzyme (ACE) gene. Pioneering work observed a link between the insertion/deletion (I/D) polymorphism in the *ACE* gene and salt-sensitive HTN, in which patients with II (Insertion/Insertion) or DI (Deletion/Insertion) genotypes were reported to have a significantly higher prevalence of salt-sensitive HTN in comparison to those having the DD (Deletion/Deletion) genotype (Giner et al., 2000). AGTR1, which codes for angiotensin receptor type 1, was nominally associated with salt-sensitive HTN phenotypes (Iwai et al., 2007). AGT gene codes for angiotensinogen interact with renin to produce angiotensin I. Mutations in the AGT gene result in the replacement of methionine by threonine eat residue 235 of the mature protein. Hence, three genotypes are detected among the population: homozygous AGT 235 TT and AGT 235MM, and heterozygous AGT 235 MT (ElAlfy et al., 2019). The mutated forms of angiotensinogen, AGT 235 TT and AGT 235 MT are unlikely to act as an early genetic predictor of salt-sensitivity, while there is a link between AGT 235 MM and salt-sensitive HTN.

#### 6.1.2 Genes-related mineralocorticoids and aldosterone

If some genes were involved in the salt-sensitivity of HTN, a good candidate would be any gene related to aldosterone and other mineralocorticoids. Different mineralocorticoids have been shown to play important roles in developing salt-sensitive HTN and associated cardiovascular and renal damage (Ayuzawa and Fujita, 2021b). One polymorphism in the 11-B-Hydroxysteroid Dehydrogenase gene has been reported to be associated with salt-sensitive HTN (Alikhani‐Koupaei et al., 2007). Another genetic polymorphism that was shown to be associated with salt-sensitive HTN includes SGK1 gene variants. Data from Caucasian volunteers who had participated in the International Hypertension Pathotype (HyperPath) group suggested that two SGK1 SNVs (rs2758151 and rs9402571) are linked with salt-sensitive HTN (Rao et al., 2013). In humans, CYP3A4 and CYP3A5 may have a role in salt-sensitive HTN and could serve as biomarkers for salt-sensitive HTN (Kuang et al., 2013). Salt-sensitivity has been linked to the cytochrome P-450 3A5 gene (CYP3A5) and its variations CYP3A5*1 (expressor) and *3 (reduced expression). Carriers of the CYP3A5 *1 allele had greater BP than those with *3, but only when they ate less salt, implying that CYP3A5 variations are involved in salt-sensitivity (Eap et al., 2007). Common variants in the aldosterone synthase were not associated with HTN salt-sensitivity (Wrona et al., 2004). Therefore, one may conclude that although the RAAS system may have a role in the etiology of HTN, data on aldosterone synthase variants suggest that it is unlikely to be the primary cause of salt-sensitive HTN.

#### 6.1.3 Genes related to renal ion transport

Ion channels and transporters in the kidneys are common final routes in regulating sodium and other ion transport. Observations reported that some ENaC mutations might cause salt-sensitive HTN. In the GenSalt study carried out in rural Northern China, participants were given a 7-days low Na^+^ intervention (3 g of salt/d) followed by a 7-days high Na^+^ intervention (18 g of salt/d) while monitoring their BP. The authors concluded that BP variation in response to dietary Na^+^ modification was linked to several ENaC single nucleotide variants (SNVs) (Mutchler et al., 2021). Additionally, multiple ENaC regulatory genes, such as NEDD4L and WNK1, have been linked to HTN and salt-sensitivity (Manunta et al., 2008). Similarly, the renal chloride channels (CLC) gene polymorphisms were related to HTN salt-sensitivity. Four SNVs of CLCNKA, expressed in Henle’s thin ascending limb, were reported to be associated with salt-sensitive HTN (Barlassina et al., 2007). Equally important, is the role of K^+^ channels in regulating renal salt handling in salt-sensitive HTN. To demonstrate the role of Kir5.1 channel encoded by Kcnj16 gene, the gene was knocked-out in Dahl salt-sensitive (SS) rats (SSKcnj16−/−). SSKcnj16−/− rats exhibited hypokalemia and reduced blood pressure and died after few days when fed high salt diet (4% NaCl) (Palygin et al., 2017). Interestingly, Benzamil protected these mice from death when fed high salt diet (Palygin et al., 2017). At this level, it is interesting to discuss is the relation between Kir5.1-mediated K+ transport and the RAAS. Although this relation remains unclear, Staruschenko et al. reported that SSKcnj16−/− rats had markedly altered RAAS regulatory hormones particularly when exposed to changes in dietary sodium and potassium content (Manis et al., 2019).

#### 6.1.4 Atrial natriuretic peptide

Many authors have researched the relationship between *ANP* gene promoter variants and the development of HTN (Rubattu et al., 2007). Several studies have suggested that ANP is important for sodium balance homeostasis and the pathophysiology of salt-sensitive HTN. An approach to investigating the genetic anomalies of ANP uncovered a link with hypertensive salt-sensitivity (Elijovich et al., 2016). In black salt-sensitive hypertensive individuals, secretion of ANP may be reduced in response to high salt diets. Similarly, homozygous deletion of the ANP gene in animals causes HTN in response to a high-salt diet, biventricular hypertrophy and cardiomyocyte swelling regardless of BP (Mishra et al., 2018). In Dahl salt-sensitive rats, deletion of the ANP gene leads to elevated BP, cardiac fibrosis, kidney hypertrophy and glomerular injury scores as well as reduced sodium and chloride excretion, upon exposure to a high salt diet (Ilatovskaya et al., 2022). These findings demonstrate that a decrease in ANP expression causes salt-sensitive HTN.

#### 6.1.5 Klotho gene

Klotho protein is mainly expressed in the kidney, but it has also been identified in the placenta, ovary, testis, aorta, and the parathyroid gland (Ben-Dov et al., 2007; Wang and Sun, 2009; Ritter et al., 2015; Olauson et al., 2017; Xiao et al., 2019). It is an obligatory coreceptor of fibroblast growth factor 23, and an important inhibitor of insulin/IGF-1 (insulin-like growth factor 1) and WNT (wingless-related integration site) signaling pathways and SIRT1 (Sirtuin1) activity. Its protective effects include stimulating NO generation and suppressing aldosterone production, apoptosis, oxidative stress, and fibrosis (Xu and Sun, 2015; Xiao et al., 2019).

Circulating Klotho levels are reduced in humans older than 40 years and patients with severe age-related diseases (Yamazaki et al., 2010; Zhou and Wang, 2015; Takenaka et al., 2018). Importantly, loss of klotho is one of the earliest impairments in CKD, a leading cause of HTN (184), occurring even in patients with a preserved GFR (CKD category G1) (Hu et al., 2011). Moreover, serum klotho levels and salt-sensitivity are inversely proportional in hypertensives (Yamazaki et al., 2010; Zhou et al., 2015; Chen and Sun, 2018). From that perspective, evidence demonstrates an important association between SNVs in the KL gene and HTN (Wang et al., 2010; Nzietchueng et al., 2011; Gao et al., 2015) and kidney disease (Haruna et al., 2007). Zhou X et al. found that transgenic mice with a one-half deficiency in the Klotho gene (KL [+/-]) exhibited an increase in BP and salt-sensitive HTN in response to excessive sodium intake (Zhou et al., 2015). More recently, a study reported that kidney injury linked with decreased Klotho levels might result in plasma aldosterone upregulation, supporting its function in salt-sensitive HTN (Qian et al., 2018). Other reports demonstrated an important role of Klotho deficiency in developing vascular aging, endothelial dysfunction, and renal immune cell infiltration, all of which contribute to HTN (Yamazaki et al., 2010; Zhou et al., 2015; Chen and Sun, 2018). Exposure of *Kl* ^+/−^ mice to high salt increased renal MCP-1 expression and subsequent macrophage and T cell infiltration, resulting in BP elevation. Inhibition of the MCP-1 receptor CCR2 (CC chemokine receptor 2) with INCB3284 attenuated these effects (Zhou et al., 2015). In 2020, Citterio et al. aimed to study the Klotho gene polymorphisms and *α*-Klotho serum levels in salt-sensitive hypertensives. They concluded that the common missense SNPs in the klotho gene (rs9536314) are linked to salt-sensitive HTN in treatment-naive patients.

Furthermore, circulating *α*-Klotho levels were primarily linked to diastolic BP alterations at the end of a salt load (Citterio et al., 2020). In light of the above, preclinical studies on multiple animal models of HTN investigated the therapeutic efficacy of Klotho supplementation. Two recent studies demonstrated that Klotho supplementation improved renal blood flow and decreased both BP and circulating and renal Ang II levels due to inhibition of WNT5a/RhoA pathway signaling (Takenaka et al., 2018; Kawarazaki et al., 2020). Klotho supplementation also enhanced pressure natriuresis through inhibition of HIF (hypoxia-inducible factor)-1α, and reduced kidney hypertrophy through inhibition of Akt-mTOR (mammalian target of rapamycin) signaling (Takenaka et al., 2018). BP and albuminuria were also reduced in db/db diabetic mice and DBA/2-pcy polycystic kidney disease mice upon klotho supplementation (Takenaka et al., 2019; Takenaka et al., 2020). Because of the critical role of Klotho in HTN pathophysiology, future studies investigating the efficacy of Klotho supplementation in humans are necessary to move forward toward modalities of therapy that increase Klotho expression.

### 6.2 Genes related to increased vascular reactivity

Multiple genes affect the physiological mechanisms that are involved in regulating BP. Despite prior research strongly indicating that primary vascular malformations play a role in the development of HTN, there are limited investigations on the role of genetic anomalies in the overall increase in BP. Genetic investigations of vascular reactivity would allow for identifying gene-affected pathways and a better understanding of salt-sensitive HTN. Therefore, in the present section, we have attempted to address some genes related to salt-sensitive HTN by increasing vascular reactivity.

#### 6.2.1 solute carrier family 24 member 3

The SLC24A3 gene in humans has been identified as a predisposition gene in salt-sensitive hypertensive individuals. Armando et al. clearly stated that SLC24A3 polymorphisms are associated with HTN salt-sensitivity. Molecularly, SLC24A3 codes for the NCKX3 protein, which maintains intracellular calcium homeostasis (Yang et al., 2017). Therefore, SLC24A3 variants alter vascular tone by increasing cytoplasmic calcium concentration (Armando et al., 2015). In agreement with that, a two-stage genetic analysis study on hypertensive patients phenotyped for their salt-sensitivity showed an interaction between SLC24A3 SNVs, pressure-natriuresis, and cellular calcium concentration. Therefore, Citterio et al. concluded that SLC24A3 polymorphisms affect the vasculature tone by altering calcium concentrations and may significantly contribute to salt-sensitive HTN development (Citterio et al., 2011).

#### 6.2.2 Solute carrier family 8 member 1

Limited research investigates the link between SLC8A1 SNVs and salt-sensitivity. SLC8A1 gene codes for the NCX1protein, which is involved in the biochemical regulation of peripheral vascular resistance. Previously, SCL8A1 has been investigated as a key molecule in the etiology of HTN and salt-sensitivity (Blaustein et al., 2009). As an extension to these findings, two SLC4A5 variants (rs7571842 and rs10177833) demonstrated a strong association with salt-sensitivity of BP in two independent Caucasian populations (Carey et al., 2012).

#### 6.2.3 Endothelin receptor type B gene

One system that controls vascular reactivity is the ET system, in which endothelin vasoactive isopeptides interact with ET_A_ and ET_B_ receptors (ET_A_R and ET_B_R). Both receptors are found in the vascular smooth muscle cells, and their activation mediates vasoconstriction (Wu et al., 2016). Additional functions have been attributed to ETBR including vasodilation via nitric oxide when expressed on endothelial cells, clearance of circulating ET-1, and regulating renal Na^+^ excretion (Caprioli et al., 2008). Although ET_A_R has shown some importance in the pathogenesis of HTN in rats, ET_B_R was reported to be associated with salt-sensitive HTN. It has been reported that deletion of ET_B_R is associated with HTN salt-sensitivity (Sanada et al., 2011). Some studies indicated that ENDRB gene polymorphism is related to the pathogenesis of salt-sensitive HTN. For example, ET_B_R 1065AA + GA (rs5351) was more frequent in salt-resistant hypertensives, demonstrating that the A allele plays a protective role. Interestingly, combining the ENDBR GG and ACE DD/ID genotypes, resulted in a synergistic effect toward salt-sensitive HTN (Caprioli et al., 2008).

#### 6.2.3 Chloride voltage-gated channel 6

Rare coding mutations and intronic SNPS in CLCN6 gene have been linked to decreased risk of HTN and stroke in recent genome-wide association studies (Flister et al., 2013). CLCN6 gene encodes for chloride channel (ClC7) which has wide range of physiological functions including the regulation of intracellular Cl^−^ concentrations (Jentsch and Pusch, 2018). After 10 days on a high salt diet, ClC6 knockout rats with Dahl salt-sensitive background (SS-Clcn6) had decreased diastolic and mean arterial BP in comparison to their healthy littermates (Flister et al., 2013). A more recent study unraveled the cardiac and vascular changes resulting from loss of ClC-6 protein function using SS-ClC6 rats. This study concluded that ClC-6 genetic ablation reduces Golgi calcium stores, impairs vasodilation, and dysregulates intracellular calcium signaling (Klemens et al., 2021). Therefore, there is growing evidence that this gene might be a heritable modifier of salt-sensitive HTN risk.

### 6.3 Genes related to immunity and inflammation

While genomic studies have discovered SNVs in genetic loci that play a role in salt-sensitive HTN’s basic processes, such as vasoconstriction and sodium reabsorption, in the past few years they have also identified genetic loci linked to inflammation and immunology. A widely studied gene in HTN is the RAG1. In Dahl salt-sensitive rats, researchers found that deleting the RAG1 gene reduced the BP elevation caused by a high-salt diet (Mattson et al., 2013). Surprisingly, The RAG1^−/−^ rats appeared to be protected from glomerular injury, albuminuria, and renal damage (Endres et al., 2014).

Interestingly, When the ζ chain (CD247) was removed from CD3^+^ in Dahl salt-sensitive rats, the phenotype was strikingly similar to that seen in rats lacking RAG1 (Rudemiller et al., 2014). In addition, more recently, the P-selectin glycoprotein ligand-1 gene (PSGL-1) was shown to be implicated in the development of salt-sensitive HTN via vascular inflammation and damage. Molecularly, the adherence of leukocytes and endothelial cells to PSGL-1 via P-selectin or/and E-selectin may cause excessive salt-induced inflammation (Yang et al., 2018). Overall, this data implies that inflammation and immunology play a major role in salt-sensitive HTN. Few SNVs and associated genetic loci have been directly tested for their causative or mechanistic implications in HTN because they appear in intergenic and noncoding regions.

Therefore, according to Madhur et al., using technology like CRISPR-Cas9 to directly introduce polymorphisms into animal models can evaluate causal roles and determine mechanisms for SNVs reported in humans (Madhur et al., 2021). Combining this strategy with other bioinformatic methodologies would help researchers better understand the genetic basis of the pathogenesis of salt-sensitive HTN.

## 7 Gene-gene interaction in salt-sensitive hypertension

Gene-gene interactions, which may affect the same or separate physiological pathways, might create a specific phenotype. As a result, combinations of several gene variants can influence susceptibility to salt-sensitive HTN. As mentioned previously, the Angiotensin-converting enzyme (*ACE* I/D) interacts with *EDNRB* 1065 GG and *HSD11B2* G534A, resulting in a synergistic effect in promoting the salt-sensitive hypertensive phenotype (Caprioli et al., 2008). In addition, according to Citterio et al., the *SLC24A3* rs3790261 - *SLC8A1* rs434082 interaction was predominant in salt-sensitive hypertensives (Citterio et al., 2011). Several gene-gene interactions in salt-sensitive hypertensive patients were listed by Armando et al. (2015). Data by Flister et al. was the first to show several causative alleles for HTN and renal phenotypes assemble at the same HTN GWAS locus. As detailed in their study, mutations of three genes at Agtrap-Plod1 locus (Nppa, Plod 1, and Mthfr) increase HTN risk, while Agtrap and Clcn six at the same locus decreases disease susceptibility (Flister et al., 2013).

## 8 Pharmacogenomics of salt-sensitive hypertension

Genetic variations may explain some of the inter-individual variability and aberrant response to antihypertensive medications. Treatment success with diuretics, ACE inhibitors, and ARBs may be significantly affected by common gene variations. However, there is a paucity of research on the pharmacogenetic variation of BP responses, particularly in salt-sensitive HTN.

## 9 Future perspectives

As mentioned before, salt-sensitivity is an independent risk factor for cardiovascular events and mortality (Weinberger et al., 2001; Elijovich et al., 2016). Therefore, it is crucial to address salt-sensitivity as an individual condition, separate from essential HTN. The lack of appropriate diagnostic tools and incidence documentation impeded advances in understanding the underlying pathologic mechanisms of salt-sensitivity (Cobb et al., 2014; Elijovich et al., 2016). Moreover, the complexity of genome regulation and the variability of HTN contribute to the delayed progress in understanding the genetics of salt-sensitive HTN and the pharmacogenetics of aberrant responses to antihypertensive medications. One of the most pressing matters is developing a standardized diagnostic method for identifying salt-sensitive BP. The Discovery of a biomarker that can identify salt-sensitive HTN would allow researchers to perform population-wide genetic investigations and associate phenotype characteristics with prognosis and gene variants or clusters of gene variants. In animals, transgenic studies that involve knockout of genes that regulate natriuresis and produce or prevent salt-sensitive HTN may even provide some of the strongest evidence on the role of putative causative or protective genes, which is necessary to detect new therapeutic targets to better individualize patient therapy and disease management.

The appreciation of immunological memory as a key component in the pathogenesis of salt-sensitivity, has important clinical implications as hypertensive stimuli are recurrent in nature (Matthews et al., 2004). For example, repeated bouts of emotional stress or dietary oversights are typical of everyday life. This may also be pertinent to other diseases, such as preeclampsia, where short-term HTN in pregnancy increases the risk for cardiovascular events well after the gestation period. To that end, an important area for improvement is the identification of neoantigens as mediators of memory in salt-sensitive HTN. This concept has been explored before in oncology (Schumacher and Schreiber, 2015). Revolutionary breakthroughs like TCR sequencing enabled critical description of TCR repertoire and characterization of T cell population diversity. Other methods to investigate TCR diversity include TCR Spectratyping. Together, these techniques provide researchers with crucial information regarding the type of T cell clonality (monoclonal, oligoclonal, or nonspecific), transcript length frequencies, presence of dominant TCR transcript lengths, and detection of non-Gaussian distributions, which enable the characterization of precise T cell subtypes and neoantigens that precipitate HTN development.

An elegant study by Troet et al. demonstrated dominant TCR transcript lengths in Vβ3, 8.1 and 17 families of kidney CD8^+^ cells upon exposure to Ang II(141). Deep sequencing of kidney CD8^+^ TCRs identified sequences that were not shared by control mice, which was not the case for CD8^+^ TCRs obtained from the spleen and mesenteric vasculature. In fact, TCR transcript lengths in the spleen and mesenteric vasculature were normally distributed and not different from sham-treated mice, indicating a lack of clonal expansion in these organs. Taken together, the presence of TCR sequences shared by Ang II treated mice exclusively in the kidney with the skewing of selected TCR Vβ family transcript lengths strongly supports the view of the kidney being a principal site for immune cell activation and neoantigen formation in HTN. Moreover, clones shared among hypertensive mice in kidney CD8^+^ T cells exhibited low frequency. This means that specific T cell clones that were initially activated lead to inflammatory conditions that result in the recruitment of other T cell clones that are not necessarily similar to the original clones (Kawakami et al., 2005). This observation also provides important information on the nature of the neoantigen and the possibility of multiple neoantigens activating multiple T cell clones, all contributing to the hypertensive response, a phenomenon known as epitope spreading (Vanderlugt and Miller, 2002). Thus, understanding peptide sequence patterns that may provoke or inhibit a T cell response will allow the development of vaccines targeting a self-antigen or a neoantigen. To that end, an important vaccine for HTN currently being developed is the AGMG0201 angiotensin II vaccine, which was well tolerated among study subjects in its phase I/IIa trial, and demonstrated detectable antibody titer in most participants (Nakagami et al., 2022). Mechanistically, the vaccine stimulates the production of antibodies against Ang II, a self-peptide hormone, without generating a cytotoxic immune response. So far, this vaccine has shown efficacy in animals, but its BP-lowering effect on humans is yet to be investigated.

In addition to TCR sequencing, technological advances in single-cell sequencing have allowed researchers to characterize previously unclassified immune cell populations. Recent evidence demonstrates that monocytes circulate and enter and exit tissues with or without differentiation, making these cells principal orchestrators of immune system activation. In that regard, recent studies described a novel anti-inflammatory role for myeloid cells in HTN (171–173), highlighting the complexity of immune cell activation in HTN and shedding light on the importance of immunoregulatory functions in achieving “immune balance” Moreover, the role of the gut microbiome in immune cell activation has been appreciated recently. Therefore, moving forward, modern technologies such as microbial sequencing and single-cell sequencing are paramount to identifying new cellular targets for therapeutic management of HTN.

An important question stems from the role of T cells in salt-sensitive HTN: What are the driving forces behind the activation and migratory behavior of T cells from the lymphoid organs and into the circulation and target tissues? Overall, the quick and targeted response of T cells to disease relies on their regulated movement between tissues and their dynamic ability to sense a multitude of directional cues and signals from a restructured inflammatory environment. (Fowell and Kim, 2021). Therefore, studies using advanced tracking techniques demonstrating T cell movement in and out of the bone marrow are crucial for understanding T cell activation and migration patterns in HTN (50). For example, the Sphingosine-1-phosphate (S1P) is bioactive lipid second messenger that been studied by our group and others, as a chief regulator of T cell escape from lymphoid organs (Aoki et al., 2016). An inhibitor of SIP-SIPR1 signaling, FTY720, has been approved for the treatment of multiple sclerosis (Prager et al., 2015). FTY720 promotes immune suppression through inhibition of lymphocyte egression from the bone marrow (Mandala et al., 2002), and is an interesting target of salt-sensitivity research.

The role of organ-specific progenitor/stem cells in salt-sensitive HTN has also been a focus of recent studies (Hu et al., 2014). Physiologically, stem cells are required for standard organ repair and maintenance (Oliver, 2004; Haller et al., 2005), and stem cell impairments have been detected in multiple diseases (Raaijmakers and Scadden, 2008; Stout and Blasco, 2009; Wilschut et al., 2012; Lagali et al., 2013). In this regard, a Hu J. et al., identified a medullary stem cell deficiency in Dahl salt-sensitive rats, that contributed to their hypertensive phenotype (Hu et al., 2014). Transplantation of mesenchymal stem cells (MSCs) into the renal medulla of these animals, ameliorated HTN, decreased sodium retention and medullary levels of MCP-1 and IL-1β as well as medullary immune cell infiltration, all of which are well-recognized features of salt-sensitive HTN. Moreover, high salt intake increased renal medullary levels of fibroblast growth factor 2 (FGF2), a stem cell niche factor, and stem cell marker CD133 in control rats but not in Dahl salt-sensitive rats, indicating an impairment in the adaptive response to excess salt. Chronic infusion of valproic acid (VA), a well-known anti-epileptic agent, into the renal medulla of Dahl salt-sensitive rats increased the stem cell CD133+ cells population and increased protein and mRNA expression of FGF2 (Wang et al., 2017). Treatment with VA also attenuated salt-induced increases in proinflammatory factors IL-1β and IL-6 and improved sodium natriuresis and excretion. VA inhibits histone deacetylases and regulates several essential genes for stem cell survival and differentiation (Bug et al., 2005; Dong et al., 2009; Han et al., 2011; Huang et al., 2015; Wang et al., 2015; Talwadekar et al., 2017). Currently, the full mechanism of action of MSCs is not fully understood; however, accumulating evidence demonstrates important anti-inflammatory actions of stem cells, which may explain their antihypertensive functions (Mariani and Facchini, 2012; Souidi et al., 2013). In that regard, transplantation of MSCs inhibited salt-induced activation of the NLRP3 inflammasome (Zhu et al., 2016), and its product IL-1β(384), which are known to be activated in salt-sensitive HTN (Bai et al., 2017; Zhao et al., 2018; Krishnan et al., 2019). MSCs have also been shown to stimulate Treg cell generation (Casiraghi et al., 2008), and macrophage polarization into the anti-inflammatory M2 cell (Kim and Hematti, 2009; Maggini et al., 2010) which are negatively regulated in salt-sensitive HTN (Imberti et al., 2007; Binger et al., 2015; Hernandez et al., 2015). In addition to immunomodulation, stem cell therapy protects against organ damage and supports tissue repair by promoting angiogenic, anti-apoptotic and oxidative pathways. Research suggests that one possible mechanism through which MSCs mediate these effects is the release of extracellular vesicles (EV) such as microvesicles/exosomes that contain cytokines, including granulocyte colony-stimulating factor, vascular endothelial growth factor, hepatocyte growth factor, IL-10, TGF-β, epidermal growth factor, and IGF-1, as well as mRNA/microRNA (Imberti et al., 2007; Jarvinen et al., 2008; Cantaluppi et al., 2013; Fleig and Humphreys, 2014; Thakkar et al., 2017). In this regard, recent progress has been made regarding the role of miRNAs in HTN pathology.

An important example is miRNA-429, which was shown to be stimulated by high salt as a protective mechanism against salt-induced HTN. Mechanistically, miR-429 induces mRNA decay of HIF prolyl-hydroxylase 2 (PHD2), increasing HIF-1α, which in turn activates HIF-1α-regulated antihypertensive genes in the renal medulla (Zhu et al., 2017). miR-429 was shown to be downregulated in the renal medulla of Dahl salt-sensitive rats, and *in vivo* transduction of lentiviruses expressing miR-429 in Dahl salt-sensitive rats improved pressure natriuresis, renal sodium excretion and decreased arterial BP after high salt challenge (Zhu et al., 2021). The above data provides the basis for the investigation of stem-cell therapy salt-sensitive HTN (Rodríguez-Iturbe et al., 2002b; Mattson et al., 2006; Larijani et al., 2012).

## 10 Conclusion

Overall, the relationship between salt consumption and salt-sensitivity of BP is an ongoing debate, and developments in our understanding of salt-sensitivity are massively hampered by its complex pathophysiology and the lack of a diagnostic strategy for classification of salt-sensitive hypertensive and normotensives. Here we provide a comprehensive review on the genetic and pathophysiological mechanisms governing salt-sensitive HTN and identify important gaps in the literature and potential areas of investigation.
